# An artificial cationic oligosaccharide combined with phosphorothioate linkages strongly improves siRNA stability

**DOI:** 10.1038/s41598-020-71896-w

**Published:** 2020-09-09

**Authors:** Atsushi Irie, Kazuki Sato, Rintaro Iwata Hara, Takeshi Wada, Futoshi Shibasaki

**Affiliations:** 1 Molecular Medical Research Project, Tokyo Metropolitan Institute of Medical Science, Setagaya-ku, Tokyo, 156-8506 Japan; 2 Calpain Project, Tokyo Metropolitan Institute of Medical Science, Tokyo, Japan; 3 Faculty of Pharmaceutical Sciences, Tokyo University of Science, Chiba, Japan; 4 Department of Neurology and Neurological Science, Graduate School of Medical and Dental Sciences, Tokyo Medical and Dental University, Tokyo, Japan; 5 Center for Medical Research Cooperation, Tokyo Metropolitan Institute of Medical Science, Tokyo, Japan

**Keywords:** Chemical biology, Drug discovery

## Abstract

Small interfering RNAs (siRNAs) are potential tools for gene-silencing therapy, but their instability is one of the obstacles in the development of siRNA-based drugs. To improve siRNA stability, we synthesised a double-stranded RNA-binding cationic oligodiaminogalactose 4mer (ODAGal4) and investigated here its characteristics for siRNA stabilisation in vitro. ODAGal4 improved the resistance of various siRNAs against serum degradation. The effect of ODAGal4 on siRNA stabilisation was further amplified by introduction of modified nucleotides into the siRNA. In particular, a combination of ODAGal4 and incorporation of phosphorothioate linkages into the siRNA prominently prevented degradation by serum. The half-lives of fully phosphorothioate-modified RNA duplexes with ODAGal4 were more than 15 times longer than those of unmodified siRNAs without ODAGal4; this improvement in serum stability was superior to that observed for other chemical modifications. Serum degradation assays of RNAs with multiple chemical modifications showed that ODAGal4 preferentially improves the stability of RNAs with phosphorothioate modification among chemical modifications. Furthermore, melting temperature analysis showed that ODAGal4 greatly increases the thermal stability of phosphorothioate RNAs. Importantly, ODAGal4 did not interrupt gene-silencing activity of all the RNAs tested. Collectively, these findings demonstrate that ODAGal4 is a potent stabiliser of siRNAs, particularly nucleotides with phosphorothioate linkages, representing a promising tool in the development of gene-silencing therapies.

## Introduction

RNA interference (RNAi) is a gene regulatory mechanism in which the expression of specific genes is silenced by endogenous double-stranded RNAs or by synthetic short interfering RNAs (siRNAs) consisting of oligoribonucleotide duplexes of 21–23 bases^1–3^. Although siRNA-based therapeutics have enormous potential for silencing specific genes that cannot be targeted by existing drugs, the development of siRNA-based drugs has been faced several difficulties: prevention of rapid degradation by nucleases, delivery to the target tissue, entry into the target cells, avoidance of innate immune responses and reduction of off-target effects^3,4^.

To reduce their instability, most of the developed siRNA agents carry chemical modifications in their nucleotide sugars, nucleobases, or phosphodiester linkages to prevent RNase cleavage^5–7^. For instance, phosphorothioate modification, which substitutes a sulphur atom for the non-bridging oxygen in the phosphodiester bond^8^, is a classical modification applied to antisense oligonucleotides^9^. Phosphorothioate modification is particularly effective for providing DNase resistance, and thus is commonly used in antisense oligodeoxynucleotides^8,10^; however, it is also applicable to siRNAs^9,11^. Moreover, phosphorothioate modification improves the cellular uptake of antisense oligonucleotides^9^ and siRNAs^12^ because of its hydrophobicity.

In addition to phosphodiester linkages, the ribose moieties of nucleotides are also widely targeted as sites for chemical modification. For example, 2′-*O*-methyl modification, which substitutes a methoxy group for the hydroxy group at the 2′-position of the ribose moiety^13,14^, is an effective modification for preventing RNase digestion. Locked nucleic acid (LNA)^15,16^ and 2′-deoxy-2′-fluoro nucleotides^17,18^ are also potent in supressing the cleavage of RNAs. For instance, the recently approved siRNA-based drugs givosiran^19^ and patisiran^20^ are modified by phosphorothioate linkages, 2′-*O*-methyl and 2′-deoxy-2′-fluoro nucleotides, and by 2′-*O*-methyl nucleotides, respectively^7^. However, chemical modification is not a perfect strategy for siRNA protection: extensive 2′-*O*-methyl, LNA and phosphorothioate modifications may reduce the gene-silencing activity of siRNAs^14,21,22^. Moreover, RNAs modified with extensive phosphorothioate modification might have increased cytotoxic effects^13,23^.

The degradation of a double-stranded siRNA by RNase occurs in two steps: first, dissociation of the double-stranded RNA into two single-stranded nucleotides; second, cleavage of the single strands by RNase^24,25^. In general, chemical modification aims to prevent the second step; therefore, an alternative strategy to preserve the siRNA duplex is to prevent dissociation of the double strand into single strands. RNA duplexes form an A-form helix structure in which the major grooves have higher negative potential than the minor grooves because anionic phosphates of the internucleotide bonds line the inside edge of the major grooves^26^. Thus, cationic molecules that can fit into the major grooves are expected to conserve the RNA duplex structure and thereby prevent RNA degradation. Indeed, aminoglycoside antibiotics with one or more protonated amino groups bind to RNA duplexes and stabilise them^27,28^, suggesting that cationic amino sugars have favourable properties for the stabilisation of RNA duplexes. For siRNA stabilisation, however, an excess amount of cationic carriers is required because of their weak affinity for siRNAs. As a consequence, these cationic carriers are prone to be cytotoxic due to nonspecific binding to other biomolecules including DNA, RNA and proteins^29,30^. Therefore, to stabilise siRNAs with low cytotoxicity, it is necessary to develop cationic molecules that can specifically bind to RNA duplexes with higher affinity.

**Figure 1:**
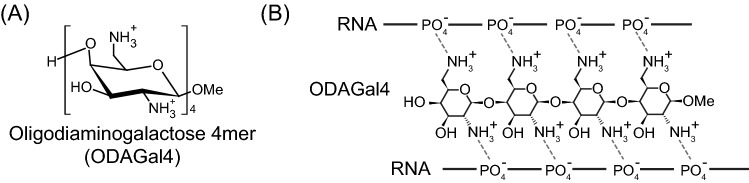
Structure of oligodiaminogalactose 4mer (ODAGal4). (**A**) The chair conformation of ODAGal4. (**B**) Schematic depiction of ODAGal4 binding to an RNA duplex.

Based on these lines of evidence, we have designed and synthesised a novel type of artificial cationic oligosaccharides with amino or guanidino groups at the 2- and 6-positions of the pyranose rings^31–33^ (Fig. 1A and Supplementary Fig. 1). The distance between the 2-*N* and 6-*N* nitrogen atoms of these cationic oligosaccharides (approximately 6 Å) is similar to the width of the major groove of A-form RNA double helix^31^, whereas that of B-form DNA double helix is much wider^34^. In addition, the saccharide backbones of these oligosaccharides can form curved structures which are favourable for binding to the RNA helix structure^31^. Therefore, these cationic oligosaccharides are expected to preferentially fit into the major groove of RNA helix and bind to the RNA duplex through interaction between the amino or guanidino groups of the oligosaccharides and the phosphate groups in the phosphodiester linkages of the RNA duplex (Fig. 1B). Indeed, we have reported that these cationic oligosaccharides bind to the major grooves of A-form RNA duplexes, but not those of B-form DNA duplexes, and consequently stabilise RNA duplexes^31–33^. Among the cationic oligosaccharide family, β-(1 → 4)-linked 2,6-diamino-2,6-dideoxy-d-galactopyranose 4mer (ODAGal4) (Fig. 1A) is the most potent siRNA stabiliser, binding to RNA duplexes more efficiently as compared with aminoglycosides, increasing thermal stability, and reducing the degradation of RNA duplexes by RNase A^33^.

Although ODAGal4 is a promising molecule for improving the stability of siRNAs, its ability to protect siRNAs that contain chemically modified nucleotides has not yet been characterised. In this study, therefore, we have investigated the effects of ODAGal4 on biological and thermal stability of RNAs with modified nucleotides. We demonstrate that a combination of ODAGal4 and phosphorothioate linkages in RNA nucleotides substantially improves the stability of siRNAs without compromising their gene-silencing activity.

## Results

### ODAGal4 increases the stability of siRNAs in serum

**Figure 2:**
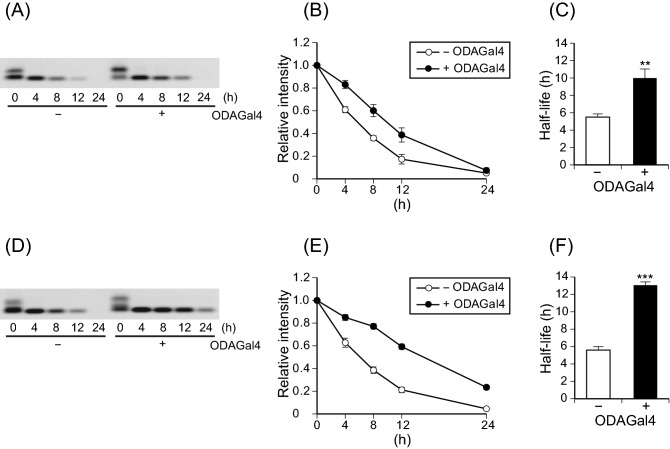
ODAGal4 increases the serum stability of HPRT1 and B2M siRNAs. siRNAs targeted to HPRT1 (HP2) (**A–C**) and B2M (B2M2) (**D–F**) were mixed with or without ODAGal4, and then incubated in 10% mouse serum at 37 °C for 0 to 24 h. The samples were separated by polyacrylamide gels and stained with a green fluorescent dye. (**A**,**D**) Representative fluorescence images of the gels. Scanned uncropped full-length gels are presented in Supplementary Fig. 2. (**B**,**E**) Fluorescence intensity of the siRNAs. Values are relative to the intensity measured at 0 h. (**C**,**F**) Half-lives of the siRNAs in serum calculated from the results in (**B**) and (**E**), respectively. Each value is the mean ± SE (n = 4).

To investigate the biological stability of siRNA, in vitro serum degradation assays are commonly used as models of siRNA digestion in body fluid because serum is easily available and contains several RNases including RNase A-type enzymes^35,36^. We prepared two siRNAs targeting hypoxanthine phosphoribosyltransferase 1 (HPRT1) and β-2-microglobulin (B2M), named HP2 and B2M2, respectively (Supplementary Table 1), and examined the effect of ODAGal4 on their stability in mouse serum. HP2 and B2M2 were completely digested within 24 h in serum in the absence of ODAGal4, with half-lives of 5.50 and 5.60 h, respectively (Fig. 2). When the HP2 and B2M2 siRNAs were mixed with four equivalents of ODAGal4 (N/P ratios, which are defined as the molar ratios between cationic amine groups in the carriers and phosphorus in the nucleotides, = 0.8), they were more resistant to degradation in serum, with half-lives of 9.98 and 13.1 h, respectively. The split bands of HP2 observed at 0 h (Fig. 2A) probably reflect non-specific interaction of HP2 with serum proteins because HP2 migrated as a single-sized band during digestion with purified RNase A, where its half-lives with or without ODAGal4 were 5.66 and 13.9 h, respectively (Supplementary Fig. 3). In addition, in a serum degradation assay of nine additional siRNAs (Supplementary Table 1), the half-lives of all siRNAs were prolonged by ODAGal4 (Supplementary Table 2), suggesting that ODAGal4 can serve as a stabiliser for various siRNAs independent of nucleotide sequence.

**Figure 3:**
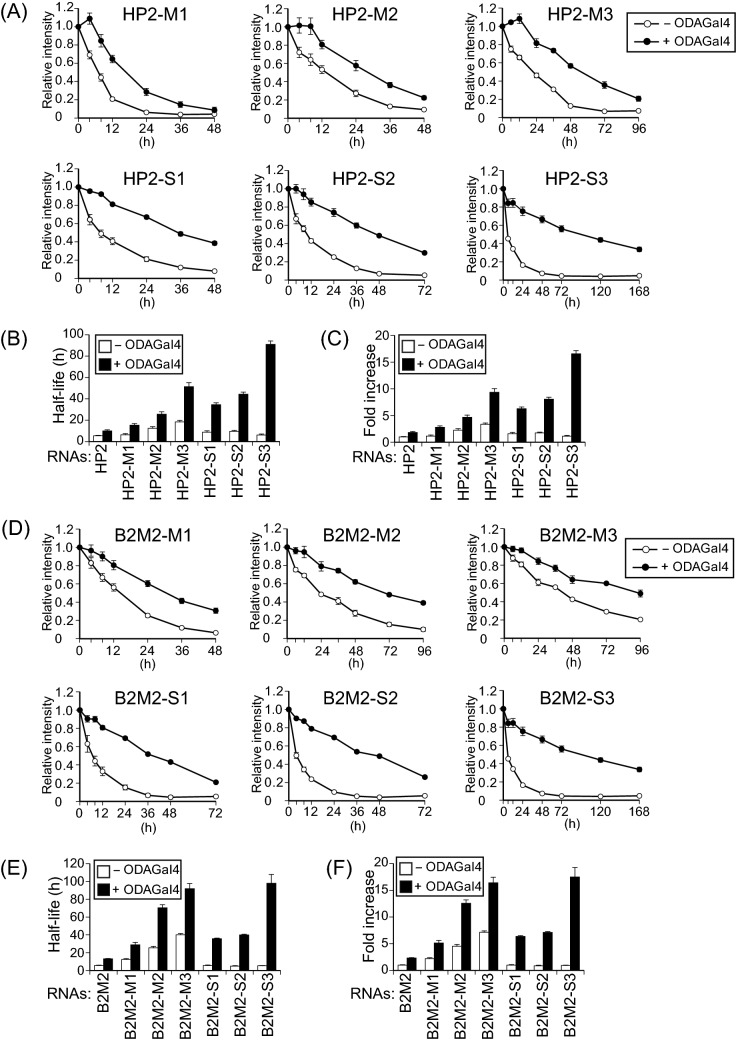
ODAGal4 improves the serum stability of 2′-*O*-methyl and phosphorothioate RNAs. 2′-*O*-Methyl or phosphorothioate HP2 (**A–C**) and B2M2 (**D–F**) with or without ODAGal4 were incubated in 10% serum for 0 to 168 h, and the remaining RNAs were quantified. (**A**,**D**) Fluorescence intensity of the RNAs. (**B**,**E**) Half-lives of the RNAs from the results in (**A**) and (**D**), respectively. (**C**,**F**) Fold increase of the half-lives quantified in (**B**) and (**E**), respectively. Each value is the mean ± SE (n = 4).

### ODAGal4 improves the serum stability of 2′-*O*-methyl and phosphorothioate RNAs

To determine the effects of ODAGal4 on the biological stabilisation of siRNAs that contains chemically modified nucleotides, we introduced 2′-*O*-methyl nucleotides into both HP2 (HP2-M1 to HP2-M3, Supplementary Table 3) and B2M2 (B2M2-M1 to B2M2-M3, Supplementary Table 4) and assessed the stability of the resulting RNAs in serum (Fig. 3). In the absence of ODAGal4, the HP2 and B2M2 with modified nucleotides showed longer half-lives than the naive HP2 and B2M2, owing to the increasing number of 2′-*O*-methyl nucleotides. When four equivalents of ODAGal4 was added to these RNAs (N/P = 0.8), their half-lives were further increased. For instance, HP2-M3 and B2M2-M3, which were introduced 2′-*O*-methyl modification into alternating nucleotides in the sense strands and on all nucleotides in the antisense strands, showed a marked improvement in serum stability, with a respective 9.34- and 16.4-fold increase in half-life as compared with the unmodified siRNAs without ODAGal4 (Fig. 3C, F).

We next prepared and assessed the serum stability of RNAs with phosphorothioate modification (HP2-S1 to HP2-S3, Supplementary Table 3; and B2M2-S1 to B2M2-S3, Supplementary Table 4). In the absence of ODAGal4, the half-lives of the phosphorothioate HP2 were slightly increased (1.12–1.60-fold increase) as compared with HP2 (Fig. 3A–C), whereas those of phosphorothioate B2M2 were not improved (0.873–1.00-fold increase) relative to B2M2 (Fig. 3D–F), suggesting that phosphorothioate modification is less effective for RNA stabilisation than 2′-*O*-methyl modification. In sharp contrast, the inclusion of ODAGal4 with the phosphorothioate RNAs prominently enhanced their stability in serum: the half-lives of RNAs modified with phosphorothioate linkages in the sense or antisense strands (HP2-S1, HP2-S2, B2M2-S1 and B2M2-S2) were longer than 30 h, corresponding to a 6.36–8.06-fold increase as compared with HP2 and B2M2 without ODAGal4. Furthermore, in the presence of ODAGal4, the RNAs completely modified with phosphorothioate linkages (HP2-S3 and B2M2-S3) were more tolerant to serum digestion than the RNAs with extensive 2′-*O*-methyl modification (HP2-M3 and B2M2-M3): the half-lives of HP2-S3 and B2M2-S3 with ODAGal4 were longer than 90 h, and the fold increases in half-life relative to HP2 and B2M2 without ODAGal4 were 16.6 and 17.5, respectively. Collectively, these results suggest that a combination of ODAGal4 and either 2′-*O*-methyl or phosphorothioate modification markedly increases the serum stability of RNAs, but the characteristics of stabilisation differ between 2′-*O*-methyl and phosphorothioate modifications.

In addition to ODAGal4, we previously developed the artificial cationic oligosaccharides α-(1 → 4)-linked 2,6-diamino-2,6-dideoxy-d-glucopyranose 4mer (ODAGlc4)^31^ and α-(1 → 4)-linked 2,6-diamino-2,6-dideoxy-d-mannopyranose 4mer (ODAMan4)^32^, whose pyranosyl structures differ from that of ODAGal4; and β-(1 → 4)-linked 2,6-dideoxy-2,6-diguanidino-d-galactopyranose 3mer (ODGGal3), which contains guanidino groups instead of amino groups^33^ (Supplementary Fig. 1). The half-life of HP2-S1 was greatly prolonged by each of the oligosaccharides (Supplementary Table 5), suggesting that the amino or guanidino groups are crucial for the marked enhancement in serum stability of the phosphorothioate RNA.

### ODAGal4 does not improve the serum stability of single-stranded RNAs

**Figure 4:**
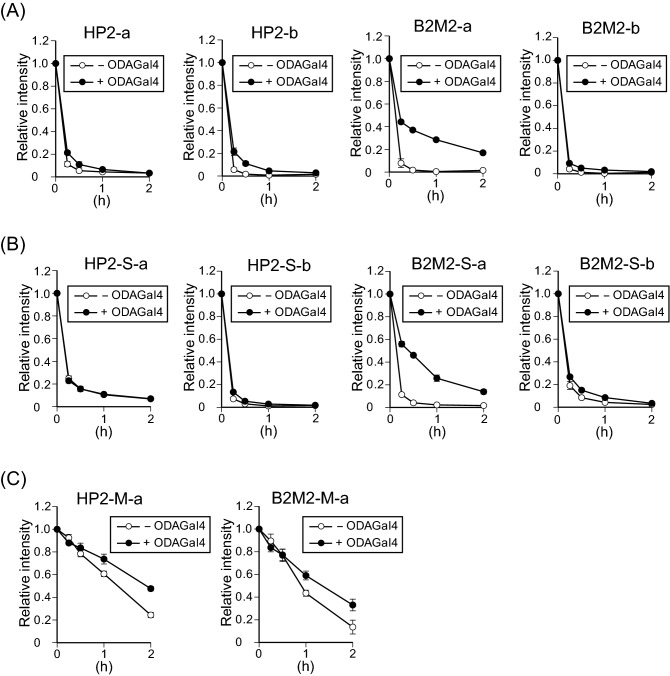
ODAGal4 does not improve the serum stability of single-stranded RNAs. Unmodified (**A**), phosphorothioate (**B**) and 2′-*O*-methyl (**C**) single-stranded oligonucleotides with or without ODAGal4 were incubated in 10% serum for 0 to 2 h, and the remaining RNAs were quantified. Each value is the mean ± SE (n = 3).

Most RNases in body fluid digest single-stranded RNAs rather than double strands^37,38^. To examine whether ODAGal4 protects single-stranded RNAs against serum digestion, the sense and antisense single strands of HP2, B2M2 and their modified analogues (Supplementary Table 6) were subjected to the serum degradation assay (Fig. 4). Unmodified HP2-a, HP2-b and B2M2-b were rapidly cleaved within 2 h with or without ODAGal4, suggesting that ODAGal4 is unable to improve serum stability of these single strands (Fig. 4A). ODAGal4 prolonged the degradation of B2M2-a because it may be able to stabilise intra- and intermolecular duplexes of the single strand (Supplementary Fig. 4). When the single strands were modified with phosphorothioate linkages, degradation of the RNAs was slightly retarded in both the presence and absence of ODAGal4 (Fig. 4B), suggesting that phosphorothioate modification provides modest protection of single-stranded RNA against serum degradation. In contrast, single-stranded RNAs with 2′-*O*-methyl modification were less susceptible to serum digestion, showing that this modification provides good protection against serum degradation (Fig. 4C). Nevertheless, ODAGal4 treatment did not alter the degradation rates of 2′-*O*-methyl single-stranded RNAs, suggesting that ODAGal4 does not improve the serum stability of unmodified and modified single-stranded RNAs unless intra- or intermolecular duplexes are formed. Furthermore, our observations indicate that the robust biological stabilisation of phosphorothioate RNAs by ODAGal4 treatment (Fig. 3) is due to the protection of double-stranded, but not single-stranded RNAs.

### ODAGal4 preferentially enhances the serum stability of RNAs partially substituted with phosphorothioate linkages in the 3′-terminal region

**Figure 5:**
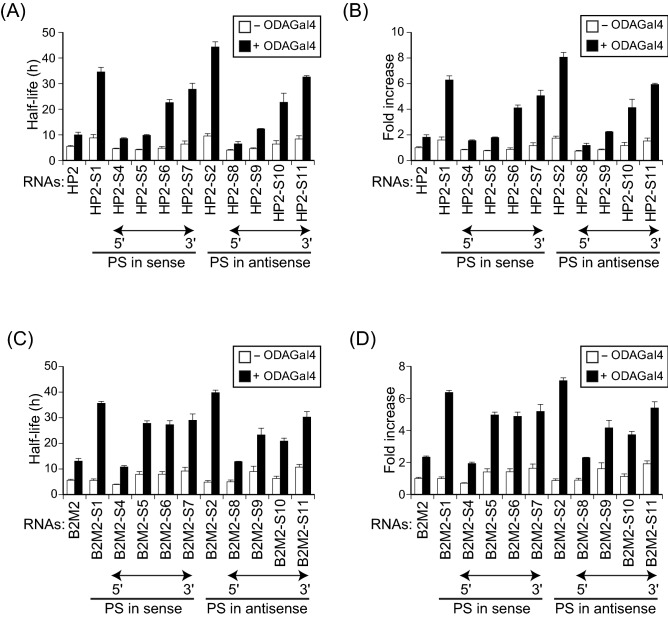
ODAGal4 preferentially enhances the serum stability of RNAs partially substituted with phosphorothioate linkages in the 3′-terminal region. HP2 (**A**,**B**) and B2M2 (**C**,**D**) partially substituted with phosphorothioate linkages were incubated in 10% serum with or without ODAGal4 for 0 to 48 h, and the remaining RNAs were quantified as shown in Supplementary Fig. 5. (**A**,**C**) Half-lives of the RNAs in serum. (**B**,**D**) Fold increase of the half-lives quantified in (**A**) and (**C**), respectively. For comparison, the results of HP2, HP2-S1, HP2-S2, B2M2, B2M2-S1 and B2M2-S2 (Fig. 3) are included. Each value is the mean ± SE (n = 4). PS, phosphorothioate linkage.

To further characterise the biological stabilisation of phosphorothioate RNAs by ODAGal4, we prepared HP2 and B2M2 that were partially modified with phosphorothioate linkages in five sequential phosphodiester linkages from the 5′- to the 3′-terminus (HP2-S4 to HP2-S11, Supplementary Table 3; and B2M2-S4 to B2M2-S11, Supplementary Table 4). In the absence of ODAGal4, the serum stability of HP2 partially modified in the sense (HP2-S4 to HP2-S7) and the antisense strand (HP2-S8 to HP2-S11) gradually improved as the position of phosphorothioate linkages shifted from the 5′- to the 3′-terminus (Fig. 5A, B, and Supplementary Fig. 5A). In the presence of ODAGal4, the serum stability of these partially modified RNAs was prominently amplified, and the positional gradients of stability were retained. Consequently, HP2-S7 and HP2-S11 with ODAGal4 showed the greatest stability: their half-lives in serum reached 27.8 and 32.6 h, respectively. Similarly, phosphorothioate modification in the 3′-terminal region was effective for stabilisation of the phosphorothioate B2M2 with or without ODAGal4 (Fig. 5C, D, and Supplementary Fig. 5B), confirming the positional preference of phosphorothioate modification in the 3′-terminus for RNA stabilisation in serum. These results indicate that phosphorothioate modification in the 3′-terminal region promotes biological stabilisation of RNAs, and addition of ODAGal4 boosts this effect.

### ODAGal4 preferentially enhances the serum stability of RNAs with phosphorothioate linkages

**Figure 6:**
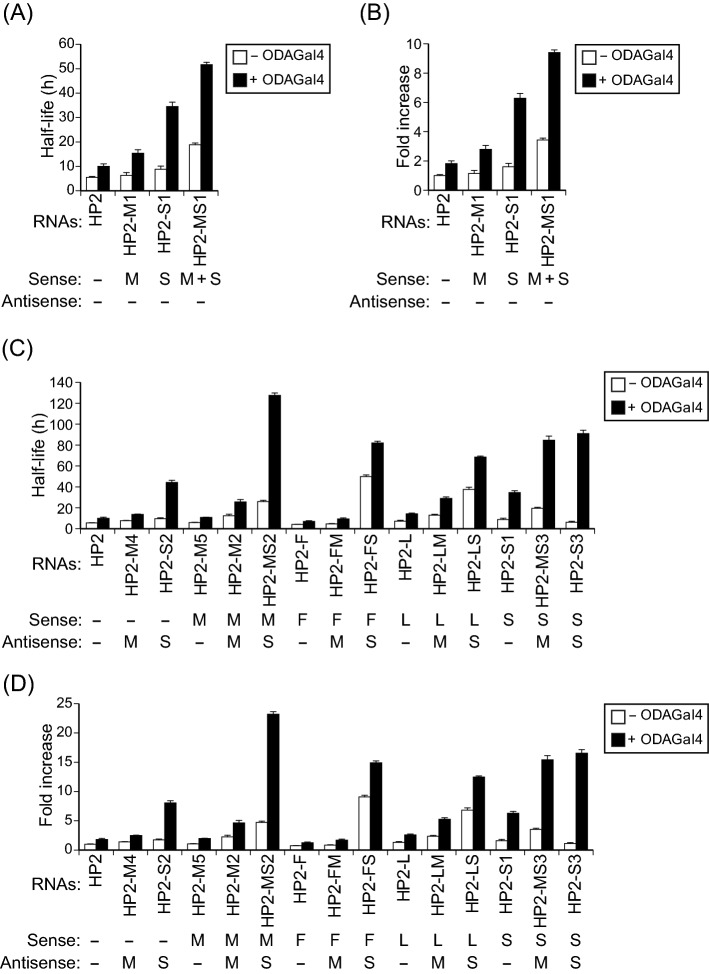
ODAGal4 preferentially enhances the serum stability of RNAs with phosphorothioate linkages. HP2 with combined modifications in the sense strands (**A**,**B**), and in both the sense and antisense strands (**C**,**D**) were prepared. The RNAs were incubated in 10% serum with or without ODAGal4 for 0 to 168 h, and the remaining RNAs were quantified as shown in Supplementary Fig. 6. (**A**,**C**) Half-lives of the RNAs in serum (n = 4). (**B**,**D**) Fold increase of the half-lives quantified in (**A**) and (**C**), respectively. For comparison, the results of HP2, HP2-M1, HP2-M2, HP2-S1, HP2-S2 and HP2-S3 (Fig. 3) are included. Each value is the mean ± SE (n = 4). F, 2′-deoxy-2′-fluoro; L, LNA; M, 2′-*O*-methyl; S, phosphorothioate modification.

It has been suggested that combinations of multiple modifications in siRNAs are more effective than single modifications for protection against cleavage in serum^39,40^. To examine whether ODAGal4 can stabilise RNAs with combined modifications, we prepared RNAs with multiple modifications (Supplementary Table 3) and observed their stability in serum (Fig. 6 and Supplementary Fig. 6). HP2 modified by both 2′-*O*-methyl nucleotides and phosphorothioate linkages in the sense strand (HP2-MS1) was more resistant to degradation than HP2 with individual modifications (HP2-M1 and HP2-S1), and addition of ODAGal4 greatly amplified its serum stability (Fig. 6A, B). Next, we assessed the serum stability of RNAs comprising a sense strand with a single modification and an antisense strand with a different modification (Fig. 6C, D). RNAs composed of a 2′-*O*-methyl, 2′-deoxy-2′-fluoro or LNA modified sense strand, and a 2′-*O*-methyl modified antisense strand (HP2-M2, HP2-FM and HP2-LM) showed a modest increase in stability with or without ODAGal4 relative to HP2. In contrast, RNAs with phosphorothioate modification on the antisense strand combined with 2′-deoxy-2′-fluoro or LNA modification on the sense strand (HP2-FS and HP2-LS) showed greatly improved serum stability even in the absence of ODAGal4. RNAs with 2′-*O*-methyl and phosphorothioate modifications (HP2-MS2 and HP2-MS3) also showed improved serum stability. These results suggest that a combination of modifications including phosphorothioate modification and other modifications is effective for biological stabilisation of RNAs. Adding ODAGal4 to the phosphorothioate RNAs further increased their half-lives: relative to HP2 without ODAGal4, HP2-MS2 and HP2-MS3 showed prominent serum stability (23.2- and 15.4-fold increase in half-life, respectively), followed by HP2-FS and HP2-LS (14.9- and 12.5-fold increase, respectively). These results demonstrate that ODAGal4 preferentially improves the biological stability of RNAs with phosphorothioate modification among chemical modifications, and that ODAGal4 and phosphorothioate modification combined with other modifications are strongly effective for siRNA stabilisation.

**Table 1:** ODAGal4 increases the thermal stability of RNAs.

Name	*T*_m_ (°C)		Δ*T*_m_ (°C)
	− ODAGal4	+ ODAGal4	(*B*–*A*)
	(*A*)	(*B*)	
HP2	79.1	80.2	1.1
HP2-M1	82.9	84.2	1.3
HP2-M2	83.0	84.9	1.9
HP2-M3	85.5	85.6	0.1
HP2-S1	75.1	78.7	3.6
HP2-S2	79.1	81.2	2.1
HP2-S3	72.1	76.7	4.6
HP2-S4	77.4	80.0	2.6
HP2-S7	78.6	81.4	2.8
B2M2	73.8	77.5	3.7
B2M2-M1	80.6	83.8	3.2
B2M2-M2	81.6	85.2	3.6
B2M2-M3	82.5	84.9	2.4
B2M2-S1	70.8	77.2	6.4
B2M2-S2	73.1	79.6	6.5
B2M2-S3	70.7	78.6	7.9

HP2 and B2M2 containing modified nucleotides (2.5 µM) were mixed with ODAGal4 (0 or 10 µM), and *T*_m_ values were analysed. Δ*T*_m_ indicates the shift in *T*_m_ value for each RNA due to ODAGal4 treatment.

### ODAGal4 increases the thermal stability of RNAs with phosphorothioate linkages

To determine whether ODAGal4 increases thermal stability of RNAs with chemically modified nucleotides, we next analysed the melting temperature (*T*_m_) of the RNAs (Table 1 and Supplementary Figs. 7 and 8). Four equivalents of ODAGal4 (N/P = 0.8) increased the *T*_m_ of unmodified HP2 and B2M2 (Δ*T*_m_ = 1.1 and 3.7 °C, respectively), in agreement with our previous study^33^. In the absence of ODAGal4, the *T*_m_ values for 2′-*O*-methyl RNAs (HP2-M1 to HP2-M3 and B2M2-M1 to B2M2-M3) were higher than those for HP2 and B2M2, suggesting that the introduction of 2′-*O*-methyl modification enhances the thermal stability of RNA duplex structures as consistent with a previous report^41^. The *T*_m_ values for the 2′-*O*-methyl RNAs were further increased by ODAGal4 treatment (Δ*T*_m_ = 0.1–3.6 °C), indicating that ODAGal4 and this modification additively enhance the thermal stability of the RNAs. In sharp contrast, the *T*_m_ values for phosphorothioate RNAs (HP2-S1 to HP2-S3 and B2M2-S1 to B2M2-S3) without ODAGal4 were lower than those for the unmodified siRNAs in good agreement with previous studies^15,42,43^. However, the *T*_m_ values for the RNAs containing modified nucleotides were markedly elevated in the presence of ODAGal4 (Δ*T*_m_ = 2.1–7.9 °C), indicating that, although phosphorothioate modification decreases the thermal stability of RNAs, the stability of RNAs is profoundly improved when this modification is combined with ODAGal4.

**Figure 7:**
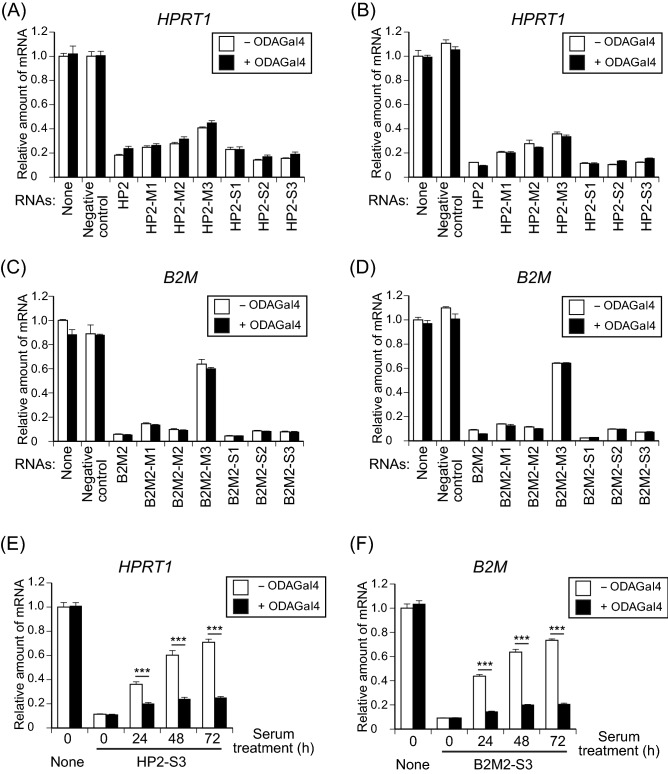
Combinations of ODAGal4 and RNAs with modified nucleotides retain gene-silencing activity. (**A**–**D**) ODAGal4 treatment retains gene-silencing activity of RNAs. HP2 (**A**,**B**) and B2M2 (**C**,**D**) containing chemically modified nucleotides (2.5 pmol) with or without ODAGal4 (10 pmol) were mixed with a lipofection reagent and transfected into Hep3B (**A**,**C**) and HeLa (**B**,**D**) cells for 4 h in serum-free medium. The transfection complexes were then removed, and the cells were cultured in medium containing 10% FBS for 2 days. Total RNAs from the cells were reverse-transcribed and subjected to quantitative PCR. Gene expression of HPRT1 (**A**,**B**) and B2M (**C**,**D**) in cells transfected with the indicated RNAs was quantified. (**E**,**F**) ODAGal4 protects the gene-silencing activity of phosphorothioate RNAs against serum degradation. HP2-S3 (**E**) and B2M2-S3 (**F**) (2.5 pmol) with or without ODAGal4 (10 pmol) were incubated in 10% mouse serum for 0 to 72 h. After incubation, the RNAs were mixed with a lipofection reagent, and transfected into HeLa cells for 4 h. The transfection complexes were then removed, and the cells were cultured for 2 days. Gene expression of HPRT1 (**E**) and B2M (**F**) in the transfected cells was quantified. Each value is the mean ± SE (n = 4).

Above, we showed that RNAs partially modified with phosphorothioate linkages in the 3′-terminal region are more stable in serum than those modified in the 5′-terminus (Fig. 5). Therefore, we next determined the *T*_m_ values for HP2-S4 and HP2-S7, which were partially modified in the 5′- and the 3′-terminus, respectively (Supplementary Table 3). Although HP2-S7 was more stable than HP2-S4 in serum, the two RNAs had similar *T*_m_ values in both the presence and absence of ODAGal4 (Table 1), showing that there is no apparent difference in thermal stability between them.

To examine whether the thermal stabilisation of RNAs by ODAGal4 is attributed to conformational changes in the RNA duplex, circular dichroism (CD) spectra of the RNA duplexes were recorded (Supplementary Fig. 9). The spectra of unmodified, 2′-*O*-methyl and phosphorothioate RNAs showed the characteristics of a typical A-form helix (i.e., a positive peak near 265 nm and a negative peak near 210 nm). The presence of ODAGal4 did not apparently alter the shape of the spectra, suggesting that ODAGal4 does not change the conformation of the RNA duplex.

We next assessed the strength of binding affinity between ODAGal4 and RNA duplexes. Because one 21mer of siRNA binds three ODAGal4 molecules^33^, we considered that the mode of ODAGal4 binding to siRNAs might be very complicated. To simplify the analysis, therefore, we prepared a 12mer RNA duplex (12M-1) that binds one ODAGal4 molecule^33^ and its analogue with phosphorothioate linkages (12M-S1) (Supplementary Table 7), and confirmed that, in the presence of ODAGal4, 12M-S1 was more resistant to serum degradation than 12M-1 (Supplementary Fig. 10A). Based on titration of ODAGal4 into 6-carboxyfluorescein (FAM)-labelled 12M-1 and FAM-12M-S1, fluorescence anisotropy determined *K*_d_ values of 0.023 and 0.012 µM, respectively (Supplementary Fig. 10B), indicating that phosphorothioate modification increases the binding affinity between RNA duplexes and ODAGal4.

### Combinations of ODAGal4 and RNAs with modified nucleotides retain gene-silencing activity

siRNAs introduced into their target cells must dissociate into single strands to induce gene-silencing activity^3^. This raises the concern that the tight interaction of ODAGal4 and siRNAs might inhibit gene-silencing activity by preventing the interaction of the RNA duplexes with the RNA-induced silencing complex (RISC), which cleaves the target mRNA for gene suppression^4^. To assess whether ODAGal4 treatment interrupts gene-silencing activity, complexes of ODAGal4 and RNAs were introduced into cultured cells and the expression of their target genes was analysed by quantitative PCR (Fig. 7). When naive HP2 or HP2 with modified nucleotides were transfected into Hep3B and HeLa cells in the absence of ODAGal4, the expression of HPRT1 was decreased by 60–80% (Fig. 7A, B). The 2′-*O*-methyl RNAs were less effective because this modification may disturb gene-silencing activity^39,44,45^. In contrast, the phosphorothioate RNAs showed effective gene silencing, indicating that phosphorothioate modification is superior to 2′-*O*-methyl modification in allowing the downregulation of gene expression. When four equivalents of ODAGal4 was mixed with the RNAs (N/P = 0.8) before transfection, the knockdown efficiencies of the siRNAs were found to be more or less comparable to those without ODAGal4 (Fig. 7A, B), indicating that ODAGal4 has marginal effect on the gene-silencing activity of siRNAs. In a similar manner, gene suppression by variously B2M2 with modified nucleotides was not affected by ODAGal4 treatment (Fig. 7C, D), confirming that complex formation between ODAGal4 and RNAs does not interrupt gene-silencing activity. Cell viability of HeLa cells transfected with HP2 and B2M2 containing modified nucleotides in the presence or absence of ODAGal4 was not decreased as compared with the untreated cells (Supplementary Fig. 11), suggesting that the reduction of gene expression levels by the RNAs coupled with ODAGal4 is not due to cytotoxicity of the RNAs nor ODAGal4.

In addition, in the presence of ODAGal4, HP2-S3 and B2M2-S3 incubated in serum for 72 h showed sufficient gene-silencing activity in HeLa cells, whereas without ODAGal4 these RNAs failed to knockdown gene expression (Fig. 7E, F). These results suggest that addition of ODAGal4 to phosphorothioate RNAs can sustain their gene-silencing activity after long-term serum treatment. Taken together, our results demonstrate that a combination of ODAGal4 and phosphorothioate linkages improves siRNA stability without compromising the gene-silencing activity of siRNAs.

## Discussion

In this study, we have shown that ODAGal4, a cationic binder of RNA duplexes, stabilises siRNAs, particularly those with phosphorothioate linkages. Phosphorothioate modification greatly improves the stability of DNA by increasing its resistance to DNase cleavage, and thus is widely used in DNA-based antisense oligonucleotides^8,10^. By contrast, this modification does not noticeably protect RNA duplexes against serum digestion^15,39^, and is probably less effective for siRNA stabilisation^15^. Our present study has shown that phosphorothioate modification modestly improves the biological stability of single-stranded RNAs in serum, but 2′-*O*-methyl modification effectively slows the degradation of RNAs. In addition, whereas 2′-*O*-methyl, LNA and 2′-deoxy-2′-fluoro nucleotides greatly increase the thermal stability of RNA duplexes^15,16,18^, phosphorothioate modification does not enhance thermal stability, indicating that phosphorothioate modification per se does not effectively improve siRNA stability. In marked contrast, we have shown that the thermal stability of phosphorothioate RNAs is strongly increased by the addition of ODAGal4, indicating that ODAGal4 remedies the thermal destabilisation caused by phosphorothioate modification. In addition, ODAGal4 greatly prolongs the half-lives of phosphorothioate RNAs in serum: in its presence, the serum stability of these nucleotides surpasses that of RNAs with other chemical modifications. We have thus demonstrated that the combination of ODAGal4 and phosphorothioate modification has great potential to improve the biological and thermal stability of siRNAs.

We previously reported that ODAGal4 stabilises RNA duplexes by interacting with amino groups in the saccharide and phosphates in the phosphodiester linkages of the nucleotides^33^. In the present study, we found that ODAGal4 stabilises RNAs modified with phosphorothioate linkages more effectively than unmodified siRNAs; thus, it is conceivable that ODAGal4 binds more tightly to the phosphorothioate moieties in the modified RNAs than to the phosphate groups in naive siRNAs. Although substituting sulphur for oxygen in a nucleotide increases the interaction with amino groups of other molecules^46,47^, no unifying mechanism for the enhancement of intermolecular interaction has yet been proposed. One plausible explanation for the enhanced interaction is that thiophosphoric acid is more acidic than phosphoric acid^48^, and therefore amino groups will interact more tightly with phosphorothioates than with phosphates. It is also likely that a phosphorothioate backbone in a nucleotide is more polyanionic than a phosphodiester linkage because the negative charges are more confined to the sulphur atoms^49^. Furthermore, substituting sulphur for oxygen in nucleotides possibly changes the mobility of the intermolecular ion pairs, resulting in entropic enhancement of the intermolecular interaction^46^. We thus suggest that the ionic interaction between ODAGal4 and the nucleotide backbone of an siRNA is strengthened by phosphorothioate linkages.

We also demonstrated that RNA duplexes partially substituted with phosphorothioate linkages in the 3′-terminus improve their stability in serum, and addition of ODAGal4 further amplifies the stability. At present, it is unclear why the position of phosphorothioate modification in the RNAs is associated with biological stability. The two RNAs that were partially modified with phosphorothioate linkages in either the 5′- or 3′-terminal region had similar *T*_m_ values in the presence and absence of ODAGal4, suggesting that the exact position of phosphorothioate linkages is less critical for thermal stability. It therefore seems likely that the effect of phosphorothioate position on biological stability of siRNAs is related to the prevention of degradation by RNase. As an endonuclease, RNase A recognises partially dissociated regions in the RNA duplex^25^; it also possesses exonuclease activity at the 3′-terminus of RNAs^50^. Thus, it is possible that phosphorothioate modification in the 3′-terminal region more effectively protects the RNAs from RNase digestion as compared with modification in the 5′-terminus. A future study to determine the precise nucleotide positions of phosphorothioate linkages in RNAs susceptible to RNase digestion will clarify the positional effects of modification on biological stabilisation of siRNAs. Notably, combined modifications including phosphorothioate modification and another modification robustly enhanced serum stability of RNAs in the presence of ODAGal4. We therefore propose that a combination of ODAGal4 and phosphorothioate RNAs with multiple modifications represents a promising strategy for siRNA stabilisation. Because ODAGal4 bind to phosphorothioate linkages but not ribose moieties, it seems reasonable to assume that ODAGal4 and phosphorothioate linkages synergistically enhance the stability of siRNAs, while modifications in the ribose moieties (i.e., 2′-*O*-methyl, LNA and 2′-deoxy-2′-fluoro nucleotides) additionally increase siRNA stability independent of ODAGal4 binding.

In RNAi, siRNAs taken up by target cells are loaded onto the RISC, and then dissociate into guide and passenger strands for gene suppression^4,51,52^. If ODAGal4 binding to the siRNA is excessively tight, the siRNA will be unable to interact with the RISC, and consequently will not separate into single strands. We previously reported that ODAGal4 binds to RNA duplexes with higher affinity as compared with neomycin B^33^, one of the strongest known RNA binders among aminoglycosides^53^, raising the concern that ODAGal4 might interrupt the gene-silencing activity of siRNAs. Notably, however, the interaction of ODAGal4 and siRNAs did not inhibit the gene-silencing activity of any siRNAs tested, including the RNAs with chemically modified nucleotides, revealing that ODAGal4 binding to RNA duplexes is not too strong; accordingly, the siRNAs can be loaded onto the RISC. Taken together, these observations demonstrate that the strength of binding between ODAGal4 and siRNAs is in an appropriate physiological range: it is tight enough to stabilise the RNA duplex, and simultaneously weak enough to allow RISC formation followed by gene suppression. Our present findings indicate that hyper-modification with phosphorothioate linkages into RNAs have sufficient gene-silencing activity without causing cytotoxicity. These findings contrast with previous reports that excessive phosphorothioate modification may reduce gene-silencing activity^21^ and induce cytotoxicity^13,23^; thus, the adverse effects of this modification should be explored further. It is therefore possible that such effects depend on the conditions of gene suppression, including cell type, siRNA sequence, and the target genes. Irrespective, the combination of ODAGal4 and phosphorothioate linkages strongly improved siRNA stability in serum, even when the nucleotides were modified only in the 3′-terminal region. We thus propose that RNAs modestly modified with phosphorothioate linkages coupled with ODAGal4 may exhibit sufficient stability and gene-silencing activity, which is prone to be interrupted by severe siRNA modification^14,21,22^.

It has been reported that various gene delivery systems which consist of cationic polymers (e.g., polyethyleneimine and polyethylene glycol) and nucleic acids have been developed to stabilise oligonucleotides for gene therapy^4,5,54^. In general, higher N/P ratios of these polycation complexes enable more effective stabilisation of the nucleotides caused by stronger interaction between the carriers and the nucleotides^54^. However, cell viability is gradually decreased with the increase of N/P ratios^55–57^, due to nonspecific binding of the polycations to other biomolecules. In addition, polycation complexes with excessively high N/P ratios show low siRNA efficiency for gene-silencing by interrupting siRNA release from the complexes^58^. These studies implicate difficulties in determining balanced N/P ratios of the polycation complexes for siRNA stabilisation with sufficient siRNA activity and low cytotoxicity. In the literature, N/P ratios of 2–100 are typically required for effective stabilisation of siRNAs by polycation complexes^56,59–63^. In sharp contrast, we have shown that ODAGal4 markedly stabilises siRNAs at a lower N/P ratio of 0.8 without reducing gene-silencing activity and cell viability, suggesting that ODAGal4 is an efficient stabiliser of siRNAs relative to the other polycation complexes. A plausible explanation for the high efficiency of ODAGal4 for siRNA stabilisation is that the binding selectivity of ODAGal4 to siRNAs. ODAGal4 preferentially binds to RNA duplexes but not to DNA duplexes nor to single-stranded DNAs because the structure of ODAGal4 fits into the major groove of A-form RNA helix but not into that of B-form DNA helix^33^. We therefore suggest that the binding of ODAGal4 to RNA duplexes is owing to the structural property of ODAGal4 and also to the ionic interaction between the amino groups of ODAGal4 and the nucleotide backbone of the RNA duplexes. In contrast, the other polycations cannot discriminate between RNA and DNA duplex structures and thereby bind to both RNA and DNA nucleotides through the ionic interaction^54,62,64,65^. Given the low binding specificity, the polycation complexes require the higher N/P ratios for siRNA stabilisation, while ODAGal4 exerts effective and selective stabilisation of siRNAs with the lower N/P ratio. In addition, we have shown in the present study that the efficacy of ODAGal4 for siRNA stabilisation is further amplified by phosphorothioate substitution of siRNAs. We thus suggest that ODAGal4 has a potential advantage as improving siRNA stability and will be a promising tool for reducing total dose and frequency of administration of siRNA-based agents in future applications.

An important goal of future studies will be application of ODAGal4 for siRNA-based drugs. ODAGal4 improved the biological stability of all siRNAs tested in our experiments, strongly suggesting that its effect is independent of siRNA sequence because it does not bind to nucleobases of the RNA duplex. Moreover, as mentioned above, ODAGal4 did not interrupt the gene-silencing activity of any siRNA tested. We thus emphasise that ODAGal4 has great potential for siRNA stabilisation, being widely applicable to various siRNA-based agents.

In conclusion, we have demonstrated that the combinations of ODAGal4 and phosphorothioate linkages in RNA duplexes vastly improves siRNA stability, with effects of stabilisation superior to those of other chemical modifications and without compromising gene-silencing activity. Although in vivo studies to confirm and expand our findings are needed, the present results nonetheless provide new insight into development of siRNA-based drugs.

## Methods

### Materials

ODAGal4, ODAGlc4, ODAMan4 and ODGGal3 were synthesised as described previously^31–33^. Sense and antisense single-stranded RNAs were synthesised by Gene Design (Osaka) and Greiner Japan (Tokyo), and annealed in 30 mM HEPES–KOH, pH 7.4, 100 mM CH_3_COOK and 2 mM Mg(CH_3_COO)_2_. The sequences of double-stranded RNA 21mers used are listed in Supplementary Tables 1, 3 and 4. The sequences of double-stranded RNA 12mers are listed in Supplementary Table 7. The single-stranded RNAs are listed in Supplementary Table 6.

### Serum degradation assay

ODAGal4 (20 pmol) and double-stranded RNA oligonucleotides (5 pmol) or single-stranded RNAs (5 pmol) were mixed and then incubated at 37 °C in 10% mouse serum (Sigma-Aldrich) for various time periods. After incubation, the reaction was stopped by the addition of recombinant RNase inhibitor (10 U) (Takara Bio) and EXELDYE 6 × DNA Loading Dye (SMOBIO). In some experiments, siRNAs were digested by 7.5 µg/ml of RNase A (Thermo Fisher Scientific) in 20 mM Tris-HCl, pH 8.0, with 150 mM NaCl. The digested RNAs were then separated by a SuperSep DNA 15% native polyacrylamide gel (FUJIFILM Wako Pure Chemical) in 25 mM Tris and 192 mM glycine, stained with SYBR Green II (Takara Bio) in 0.5 × Tris borate EDTA buffer, and visualised with an LAS-4000 image analyser (FUJIFILM). The fluorescence intensity of the remaining RNAs was quantified by using ImageJ software (National Institute of Health, USA), and the half-lives of RNAs in serum were calculated by using an exponential curve-fitting algorithm in DeltaGraph (Red Rock software).

### Melting temperature analysis

Absorbance versus temperature profiles were assessed as described previously^33^. In brief, annealed double-stranded RNAs or single-stranded RNAs (2.5 µM) were mixed with ODAGal4 (0–10 µM) in 10 mM NaH_2_PO_4_–Na_2_HPO_4_ buffer, pH 7.0, containing 100 mM NaCl. UV absorbance of the RNA/ODAGal4 complexes was monitored at 260 and 320 nm with a UV-1650 PC spectrophotometer (SHIMADZU) over a temperature gradient of 0.5 °C/min from 20 to 95 °C. All the UV absorbance values at 260 nm were baseline corrected by subtracting the readings at 320 nm. The thermal melting curves were obtained by plotting the corrected UV absorbance (the A260 reading—the A320 reading) against temperature. The vertical axis was normalised by the value at 20 °C. The *T*_m_ value was determined from the peak value of the first derivative of the thermal melting curve.

### RNA secondary structure prediction

RNA secondary structure prediction was conducted by using MaxExpect^66^ and Bifold^67^ with default settings.

### CD spectroscopy

CD spectroscopy was conducted as described previously^68^. In brief, each annealed RNA (1.5 µM) was prepared in 10 mM NaH_2_PO_4_-Na_2_HPO_4_ buffer, pH 7.0, containing 100 mM NaCl (250 µL), and the CD spectrum was recorded with a J-820 CD spectropolarimeter (JASCO) at a wavelength of 200 to 320 nm at 37 °C. After measurement, 0.1 mM ODAGal4 (15 µL) was added to the sample solution, and the CD spectrum was recorded again. The instrument settings were resolution, 0.1 nm; sensitivity, 5 mdeg; response, 1 s; speed, 100 nm/min; accumulation, 20.

### Fluorescence anisotropy

Fluorescence anisotropy was conducted as described previously^33^. In brief, 5′-FAM-labelled RNA duplexes (100 nM) were titrated with 0–700 nM ODAGal4 in 10 mM NaH_2_PO_4_–Na_2_HPO_4_ buffer, pH 7.0, containing 100 mM of NaCl and 0.02% Tween 20 at 20 °C. The change in fluorescence anisotropy was monitored with a FP-6500 spectrofluorometer (JASCO) by averaging three measurements. The instrument settings were Ex/Em = 490/520 nm; response, 2 s; bandwidth (Ex), 5 nm; bandwidth (Em), 5 nm; PMT voltage, 430 V; number of cycles, 4. The dissociation constant of ODAGal4 and each RNA duplex was calculated by the method of Wang et al.^69^

### Cell culture and transfection

HeLa cells and Hep3B cells were obtained from Japanese Collection of Research Bioresources Cell Bank and American Type Culture Collection, respectively. Cells (1.5–3 × 10^4^ cells) were cultured for a day in Dulbecco's modified Eagle medium (DMEM) containing 10% (v/v) FBS in 48-well plates. For the transfection complex, RNA (2.5 pmol) was mixed with ODAGal4 (10 pmol) at room temperature, and then Lipofectamine RNAiMAX (0.75 µg) (Thermo Fisher Scientific) was added. In some experiments, RNA (2.5 pmol) with ODAGal4 (10 pmol) was incubated at 37 °C in 10% mouse serum for 0 to 72 h, and then mixed with Lipofectamine RNAiMAX (0.75 µg). The cell culture medium was replaced with 250 µL of Opti-MEM medium (Thermo Fisher Scientific), and the RNA/ODAGal4/Lipofectamine complex was added to the cells. After 4 h, the transfection complex was removed, and the cells were cultured in DMEM with 10% FBS for 2 days before gene expression analysis. The negative control siRNA was 5′-GUACCGCACGUCAUUCGUAUC-3′ (sense) and 5′-UACGAAUGACGUGCGGUACGU-3′ (antisense).

### Quantitative PCR analysis

Total RNA was extracted from cells with TRIsol reagent (Thermo Fisher Scientific), and reverse-transcribed by using PrimeScript RT Master Mix (Takara Bio) in accordance with the manufacturer’s protocol. Quantitative PCR analysis was performed with TB Green Premix Ex Taq II (Takara Bio) and a LightCycler 480 (Roche). The relative expression level of the mRNAs for B2M and HPRT1 genes was calculated by the ΔΔCt method using glyceraldehyde 3-phosphate dehydrogenase as an internal control. The oligonucleotide primers for detection were designed by using ProbeFinder software (Roche): B2M, 5′-TTCTGGCCTGGAGGCTATC-3′ and 5′-TCAGGAAATTTGACTTTCCATTC-3′; glyceraldehyde 3-phosphate dehydrogenase, 5′-AGCCACATCGCTCAGACAC-3′ and 5′-GCCCAATACGACCAAATCC-3′; HPRT1, 5′-TGACCTTGATTTATTTTGCATACC-3′ and 5′-CGAGCAAGACGTTCAGTCCT-3′. The R^2^ value for each primer pair was over 0.99, and the efficiency of PCR was between 98 and 102%.

### Cell viability assay

HeLa cells were transfected by RNA (2.5 pmol) with or without ODAGal4 (10 pmol) and cultured for 2 days at the same condition as described above. Cell viability was determined by the 3-(4,5-dimethyhiazol-2-yl)-5-(3-carboxymethoxyphenyl)-2-(4-sulphophenyl)-2H-tetrazolium (MTS) method by using CellTiter 96 Aqueous Non-Radioactive Cell Proliferation Assay kit (Promega) in accordance with the manufacturer’s protocol.

### Statistics

Data were expressed as the mean ± SE. Statistical significance was assessed by unpaired Student’s *t* test. In the figures, statistical significance is denoted as follows: *p < 0.05, **p < 0.01, and ***p < 0.001.

## Supplementary information

Supplementary Information.
