# Supplementary material for: An artificial cationic oligosaccharide combined with phosphorothioate linkages strongly improves siRNA stability
Source: Sci Rep. 2020 Sep 9;10:14845. doi: 10.1038/s41598-020-71896-w (PMC7481297; doi:10.1038/s41598-020-71896-w)
Supplement: Supplementary file 1 — Supplementary Information. [file 41598_2020_71896_MOESM1_ESM.pdf]

Supplementary information

An artificial cationic oligosaccharide combined with phosphorothioate linkages strongly improves siRNA stability

Atsushi Irie[1, 2*], Kazuki Sato[3], Rintaro Iwata Hara[3, 4], Takeshi Wada[3] and Futoshi Shibasaki[1, 5*]

[1]Molecular Medical Research Project, Tokyo Metropolitan Institute of Medical Science, Tokyo, Japan.,

[2]Calpain Project, Tokyo Metropolitan Institute of Medical Science, Tokyo, Japan.,

[3]Faculty of Pharmaceutical Sciences, Tokyo University of Science, Chiba, Japan.,

[4]Department of Neurology and Neurological Science, Graduate School of Medical and Dental Sciences, Tokyo Medical and Dental University, Tokyo, Japan.,

[5]Center for Medical Research Cooperation, Tokyo Metropolitan Institute of Medical Science, Tokyo, Japan.

*Addresses correspondence to: Atsushi Irie, PhD, Molecular Medical Research Project, Tokyo Metropolitan Institute of Medical Science, Setagaya-ku, Tokyo 156-8506, Japan. Tel: 81-3-5316-3124 ex. 2560; E-mail: irie-at@igakuken.or.jp, and Futoshi Shibasaki, MD, PhD, Molecular Medical Research Project, Tokyo Metropolitan Institute of Medical Science, Setagaya-ku, Tokyo 156-8506, Japan. Tel: 81-3-5316-3128 ex. 1731; E-mail: shibasaki-ft@igakuken.or.jp

Supplementary Figure 1. Structures of artificial cationic oligosaccharides. (A) Oligodiaminoglucose 4mer (ODAGlc4). **(B)** Oligodiaminomannose 4mer (ODAMan4). **(C)** Oligodiguanidinogalactose 3mer (ODGGal3).

Supplementary Figure 2. Serum degradation assay of HP2 and B2M2 with ODAGal4. Uncropped raw data for fluorescence images of the gels shown in Fig. 2A and D. **(A)** Degradation of HP2 by serum for Fig. 2A. **(B)** Degradation of B2M2 by serum for Fig. 2D. The red rectangles indicate the RNAs shown in Fig. 2A and D, respectively. M, 10 bp deoxynucleotide ladder marker.

Supplementary Figure 3. ODAGal4 improves the resistance of HP2 to RNase A cleavage. HP2 was mixed with or without ODAGal4, and then incubated in RNase A (7.5 μg/ml) at 37 °C for 0 to 24 h. The remaining siRNAs were separated by a polyacrylamide gel and stained. **(A)** A representative fluorescence image of the gel. M, 10 bp deoxynucleotide ladder marker. **(B)** Fluorescence intensity of the siRNAs. **(C)** Half-lives of the siRNAs from the results in **(B)**. Each value is the mean ± SE (n = 4).

Supplementary Figure 4. Secondary structures of single-stranded B2M2 oligonucleotides. (A) UV melting curves of B2M2-a and B2M2-b. UV absorbance of the oligonucleotides with or without ODAGal4 was monitored at 260 and 320 nm with a temperature gradient from 20 to 95 °C. All data were normalised between 0 (folded) and 1 (unfolded). **(B)** Prediction of B2M2-a and B2M2-b secondary structure. The sequences of the single strands were applied to the RNA secondary structure prediction programs MaxExpect and Bifold using the default settings. Dashed lines represent predicted base pairs.

Supplementary Figure 5. ODAGal4 combined with phosphorothioate linkages in the 3'-terminal region prolongs the half-life of RNAs in serum. HP2 **(A)** and B2M2 **(B)** partially modified with phosphorothioate linkages were mixed with or without ODAGal4, and then incubated in 10% serum for 0 to 48 h. After the incubation, the remaining RNAs were quantified. Each value is the mean ± SE (n = 4).

Supplementary Figure 6. ODAGal4 prolongs the half-life of RNAs with combined chemical modifications. HP2 with combined modifications were mixed with or without ODAGal4, and then incubated in 10% serum for 0 to 168 h. After incubation, the remaining RNAs were quantified. Each value is the mean ± SE (n = 4). The modifications were **(A)** 2'-*O*-methyl; **(B)** 2'-*O*-methyl and phosphorothioate; **(C)** 2'-deoxy-2'-fluoro and 2'-*O*-methyl or phosphorothioate; **(D)** LNA and 2'-*O*-methyl or phosphorothioate.

Supplementary Figure 7. UV melting curves of HP2 with modified nucleotides in the presence of ODAGal4. UV absorbance of HP2 with or without modified nucleotides was monitored at 260 and 320 nm with a temperature gradient from 20 to 95 °C in the presence or absence of ODAGal4. All data were normalised between 0 (folded) and 1 (unfolded).

Supplementary Figure 8. UV melting curves of B2M2 with modified nucleotides in the presence of ODAGal4. UV absorbance of B2M2 with or without modified nucleotides was monitored in the presence or absence of ODAGal4.

Supplementary Figure 9. CD spectra of RNAs with modified nucleotides in the presence

of ODAGal4. CD spectra of HP2 and B2M2 containing chemically modified nucleotides were monitored at wavelength of 200 to 320 nm in the presence or absence of ODAGal4.

Supplementary Figure 10. Phosphorothioate modification of the RNA duplex increases binding to ODAGal4. (A) Serum stability of a double-stranded RNA 12mer (12M-1) and its analogue with phosphorothioate linkages (12M-S1). 12M-1 and 12M-S1 with or without ODAGal4 were incubated in 10% serum for 0 to 4 h, and the remaining RNAs were quantified. Each value is the mean ± SE (n = 4). **(B)** Binding of ODAGal4 to 12M-1 and 12M-S1. ODAGal4 (0 to 700 nM) was titrated into 5'-FAM-labelled 12M-1 or 12M-S1 (100 nM) at 20 °C, and fluorescence anisotropy was measured. The K_d values for ODAGal4 binding to the duplexes were calculated from the titration curves.

Supplementary Figure 11. Viability of cells treated with ODAGal4 and RNAs with modified nucleotides. HP2 and B2M2 containing modified nucleotides (2.5 pmol) with or without ODAGal4 (10 pmol) were transfected into HeLa cells for 4 h, and then the cells were cultured for 2 days. After incubation, cell viability was determined by using MTS assay. Values are expressed as a percentage relative to the untreated control cells. Each value is the mean ± SE (n = 4).

Supplementary Table 1. Sequences of siRNAs for various target genes.

Target	Name	Sequence
HPRT1	HP1	5′-GGUCAGGCAGUAUAAUCCAAA-3′<br>3′-AACCAGUCCGUCAUAUUAGGU-5′
HPRT1	HP2	5′-CCAGUCAACAGGGGACAUAAA-3′<br>3′-CUGGUCAGUUGUCCCCUGUAU-5′
HPRT1	HP3	5′-CAGGACUGAACGUCUUGCUCG-3′<br>3′-CUGUCCUGACUUGCAGAACGA-5′
B2M	B2M1	5′-GUGGGAUCGAGACAUGUAAGC-3′<br>3′-UUCACCCUAGCUCUGUACAUU-5′
B2M	B2M2	5′-GCAAGGACUGGUCUUUCUAUC-3′<br>3′-GUCGUUCCUGACCAGAAAGAU-5′
KIF11	KF1	5′-GGAGGUUGUAAGCCAAUGUUG-3′<br>3′-AACCUCCAACAUUCGGUUACA-5′
KIF11	KF2	5′-GCGUACAAGAACAUCUAUAAU-3′<br>3′-CCCGCAUGUUCUUGUAGAUAU-5′
KIF11	KF3	5′-GAGGAGCUGAAUAGGGUUACA-3′<br>3′-UCCUCCUCGACUUAUCCCAAU-5′
APOB	APO1	5′-UAGUUGUAUGUGUACUUCCGG-3′<br>3′-GUAUCAACAUACACAUGAAGG-5′
APOB	APO2	5′-CUCGGAACUAUCAACUCUACA-3′<br>3′-GAGAGCCUUGAUAGUUGAGAU-5′
APOB	APO3	5′-UCAAUGACUCGUAAUCAGCCU-3′<br>3′-GGAGUUACUGAGCAUUAGUCG-5′

HPRT1, hypoxanthine phosphoribosyltransferase 1; B2M, β-2-microglobulin; KIF11, kinesin family member 11; APOB, apolipoprotein B.

Supplementary Table 2. ODAGal4 extends the half-life of various siRNAs in serum. Various siRNAs with or without ODAGal4 were incubated in 10% serum for 0 to 24 h. The remaining siRNAs were quantified, and their half-lives were calculated. For comparison, the results of HP2 and B2M2 (Fig. 2) are included. Each value is the mean ± SE (n = 4).

Name	Target		Half-life (h)		
			− ODAGal4 (*A*)	+ ODAGal4 (*B*)	*B*/*A*
HP1	HPRT1		5.57 ± 0.66	9.10 ± 0.83	1.64
HP2	HPRT1		5.50 ± 0.37	9.98 ± 1.05	1.82
HP3	HPRT1		7.15 ± 0.47	11.3 ± 1.3	1.58
B2M1	B2M		2.57 ± 0.06	8.05 ± 0.47	3.13
B2M2	B2M		5.60 ± 0.40	13.1 ± 0.4	2.34
KF1	KIF11		3.60 ± 0.27	8.97 ± 0.28	2.49
KF2	KIF11		2.42 ± 0.15	5.25 ± 0.18	2.17
KF3	KIF11		9.06 ± 0.74	14.3 ± 0.2	1.58
APO1	APOB		2.13 ± 0.15	5.52 ± 0.47	2.59
APO2	APOB		4.66 ± 0.21	10.6 ± 1.0	2.28
APO3	APOB		3.47 ± 0.21	5.57 ± 0.42	1.61
		Average	4.70	9.25	1.97

Supplementary Table 3. Sequences of HP2 with modified nucleotides.

Name	Sequence
HP2-M1	5′-**c.c.a.g.u.c.a.a.c.a.g.g.g.g.a.c.a.u.a.a.a**-3′<br>3′-C.U.G.G.U.C.A.G.U.U.G.U.C.C.C.C.U.G.U.A.U-5′
HP2-M2	5′-**c**.C.**a**.G.**u**.C.**a**.A.**c**.A.**g**.G.**g**.G.**a**.C.**a**.U.A.A.A-3′<br>3′-C.U.G.**g**.U.**c**.A.**g**.U.**u**.G.**u**.C.**c**.C.**c**.U.**g**.U.**a**.U-5′
HP2-M3	5′-**c.c.a.g.u.c.a.a.c.a.g.g.g.g.a.c.a.u.a.a.a**-3′<br>3′-C.U.G.**g**.U.**c**.A.**g**.U.**u**.G.**u**.C.**c**.C.**c**.U.**g**.U.**a**.U-5′
HP2-M4	5′-C.C.A.G.U.C.A.A.C.A.G.G.G.G.A.C.A.U.A.A.A-3′<br>3′-C.U.G.**g**.U.**c**.A.**g**.U.**u**.G.**u**.C.**c**.C.**c**.U.**g**.U.**a**.U-5′
HP2-M5	5′-**c**.C.**a**.G.**u**.C.**a**.A.**c**.A.**g**.G.**g**.G.**a**.C.**a**.U.A.A.A-3′<br>3′-C.U.G.G.U.C.A.G.U.U.G.U.C.C.C.C.U.G.U.A.U-5′
HP2-S1	5′-C*C*A*G*U*C*A*A*C*A*G*G*G*G*A*C*A*U*A*A*A-3′<br>3′-C.U.G.G.U.C.A.G.U.U.G.U.C.C.C.C.U.G.U.A.U-5′
HP2-S2	5′-C.C.A.G.U.C.A.A.C.A.G.G.G.G.A.C.A.U.A.A.A-3′<br>3′-C*U*G*G*U*C*A*G*U*U*G*U*C*C*C*C*U*G*U*A*U-5′
HP2-S3	5′-C*C*A*G*U*C*A*A*C*A*G*G*G*G*A*C*A*U*A*A*A-3′<br>3′-C*U*G*G*U*C*A*G*U*U*G*U*C*C*C*C*U*G*U*A*U-5′
HP2-S4	5′-C*C*A*G*U*C.A.A.C.A.G.G.G.G.A.C.A.U.A.A.A-3′<br>3′-C.U.G.G.U.C.A.G.U.U.G.U.C.C.C.C.U.G.U.A.U-5′
HP2-S5	5′-C.C.A.G.U.C*A*A*C*A*G.G.G.G.A.C.A.U.A.A.A-3′<br>3′-C.U.G.G.U.C.A.G.U.U.G.U.C.C.C.C.U.G.U.A.U-5′
HP2-S6	5′-C.C.A.G.U.C.A.A.C.A.G*G*G*A*C.A.U.A.A.A-3′<br>3′-C.U.G.G.U.C.A.G.U.U.G.U.C.C.C.C.U.G.U.A.U-5′
HP2-S7	5′-C.C.A.G.U.C.A.A.C.A.G.G.G.G.A.C*A*U*A*A*A-3′<br>3′-C.U.G.G.U.C.A.G.U.U.G.U.C.C.C.C.U.G.U.A.U-5′
HP2-S8	5′-C.C.A.G.U.C.A.A.C.A.G.G.G.G.A.C.A.U.A.A.A-3′<br>3′-C.U.G.G.U.C.A.G.U.U.G.U.C.C.C.C*U*G*U*A*U-5′
HP2-S9	5′-C.C.A.G.U.C.A.A.C.A.G.G.G.G.A.C.A.U.A.A.A-3′<br>3′-C.U.G.G.U.C.A.G.U.U.G*U*C*C*C*C.U.G.U.A.U-5′
HP2-S10	5′-C.C.A.G.U.C.A.A.C.A.G.G.G.G.A.C.A.U.A.A.A-3′<br>3′-C.U.G.G.U.C*A*G*U*U*G.U.C.C.C.C.U.G.U.A.U-5′
HP2-S11	5′-C.C.A.G.U.C.A.A.C.A.G.G.G.G.A.C.A.U.A.A.A-3′<br>3′-C*U*G*G*U*C.A.G.U.U.G.U.C.C.C.C.U.G.U.A.U-5′
HP2-MS1	5′-**c*c*a*g*u*c*a*a*c*a*g*g*g*g*a*c*a*u*a*a*a**-3′<br>3′-C.U.G.G.U.C.A.G.U.U.G.U.C.C.C.C.U.G.U.A.U-5′
HP2-MS2	5′-**c**.C.**a**.G.**u**.C.**a**.A.**c**.A.**g**.G.**g**.G.**a**.C.**a**.U.A.A.A-3′<br>3′-C*U*G*G*U*C*A*G*U*U*G*U*C*C*C*C*U*G*U*A*U-5′

HP2-MS3	5′-C*C*A*G*U*C*A*A*C*A*G*G*G*G*A*C*A*U*A*A*A-3′<br>3′-C.U.G.**g**.U.**c**.A.**g**.U.**u**.G.**u**.C.**c**.C.**c**.U.**g**.U.**a**.U-5′
HP2-L	5′-**C**.**C**.A.G.**T**.C.A.A.C.A.G.**G**.G.G.**A**.C.A.U.A.**A**.**A**-3′<br>3′-C.U.G.G.U.C.A.G.U.U.G.U.C.C.C.C.U.G.U.A.U-5′
HP2-LS	5′-**C**.**C**.A.G.**T**.C.A.A.C.A.G.**G**.G.G.**A**.C.A.U.A.**A**.**A**-3′<br>3′-C*U*G*G*U*C*A*G*U*U*G*U*C*C*C*C*U*G*U*A*U-5′
HP2-LM	5′-**C**.**C**.A.G.**T**.C.A.A.C.A.G.**G**.G.G.**A**.C.A.U.A.**A**.**A**-3′<br>3′-C.U.G.**g**.U.**c**.A.**g**.U.**u**.G.**u**.C.**c**.C.**c**.U.**g**.U.**a**.U-5′
HP2-F	5′-**c**.**c**.**a**.**g**.**u**.**c**.**a**.**a**.**c**.**a**.**g**.**g**.**g**.**g**.**a**.**c**.**a**.**u**.**a**.**a**.**a**-3′<br>3′-C.U.G.G.U.C.A.G.U.U.G.U.C.C.C.C.U.G.U.A.U-5′
HP2-FS	5′-**c**.**c**.**a**.**g**.**u**.**c**.**a**.**a**.**c**.**a**.**g**.**g**.**g**.**g**.**a**.**c**.**a**.**u**.**a**.**a**.**a**-3′<br>3′-C*U*G*G*U*C*A*G*U*U*G*U*C*C*C*C*U*G*U*A*U-5′
HP2-FM	5′-**c**.**c**.**a**.**g**.**u**.**c**.**a**.**a**.**c**.**a**.**g**.**g**.**g**.**g**.**a**.**c**.**a**.**u**.**a**.**a**.**a**-3′<br>3′-C.U.G.**g**.U.**c**.A.**g**.U.**u**.G.**u**.C.**c**.C.**c**.U.**g**.U.**a**.U-5′

Uppercase, unmodified RNA; bold lowercase, 2'-*O*-methyl RNA; underlined bold uppercase, LNA; underlined bold lowercase, 2'-deoxy-2'-fluoro RNA; asterisk, phosphorothioate linkage. **C** indicates LNA-5-methylcytosine.

Supplementary Table 4. Sequences of B2M2 with modified nucleotides.

Name	Sequence
B2M2-M1	5′-**g.c.a.a.g.g.a.c.u.g.g.u.c.u.u.u.c.u.a.u.c**-3′<br>3′-G.U.C.G.U.U.C.C.U.G.A.C.C.A.G.A.A.A. G.A.U-5′
B2M2-M2	5′-G.**c**.A.**a**.G.**g**.A.**c**.U.**g**.G.**u**.C.**u**.U.**u**.C.**u**.A.U.C-3′<br>3′-G.U.**c**.G.**u**.U.**c**.C.**u**.G.**a**.C.**c**.A.**g**.A.**a**.A.**g**.A.U-5′
B2M2-M3	5′-**g.c.a.a.g.g.a.c.u.g.g.u.c.u.u.u.c.u.a.u.c**-3′<br>3′-G.U.**c**.G.**u**.U.**c**.C.**u**.G.**a**.C.**c**.A.**g**.A.**a**.A.**g**.A.U-5′
B2M2-S1	5′-G*C*A*A*G*G*A*C*U*G*G*U*C*U*U*U*C*U*A*U*C-3′<br>3′-G.U.C.G.U.U.C.C.U.G.A.C.C.A.G.A.A.A.G.A.U-5′
B2M2-S2	5′-G.C.A.A.G.G.A.C.U.G.G.U.C.U.U.U.C.U.A.U.C-3′<br>3′-G*U*C*G*U*U*C*C*U*G*A*C*C*A*G*A*A*A*G*A*U-5′
B2M2-S3	5′-G*C*A*A*G*G*A*C*U*G*G*U*C*U*U*U*C*U*A*U*C-3′<br>3′-G*U*C*G*U*U*C*C*U*G*A*C*C*A*G*A*A*A*G*A*U-5′
B2M2-S4	5′-G*C*A*A*G*G.A.C.U.G.G.U.C.U.U.U.C.U.A.U.C-3′<br>3′-G.U.C.G.U.U.C.C.U.G.A.C.C.A.G.A.A.A.G.A.U-5′
B2M2-S5	5′-G.C.A.A.G.G*A*C*U*G*G.U.C.U.U.U.C.U.A.U.C-3′<br>3′-G.U.C.G.U.U.C.C.U.G.A.C.C.A.G.A.A.A.G.A.U-5′
B2M2-S6	5′-G.C.A.A.G.G.A.C.U.G.G*U*C*U*U*U.C.U.A.U.C-3′<br>3′-G.U.C.G.U.U.C.C.U.G.A.C.C.A.G.A.A.A.G.A.U-5′
B2M2-S7	5′-G.C.A.A.G.G.A.C.U.G.G.U.C.U.U.U*C*U*A*U*C-3′<br>3′-G.U.C.G.U.U.C.C.U.G.A.C.C.A.G.A.A.A.G.A.U-5′
B2M2-S8	5′-G.C.A.A.G.G.A.C.U.G.G.U.C.U.U.U.C.U.A.U.C-3′<br>3′-G.U.C.G.U.U.C.C.U.G.A.C.C.A.G.A*A*A*G*A*U-5′
B2M2-S9	5′-G.C.A.A.G.G.A.C.U.G.G.U.C.U.U.U.C.U.A.U.C-3′<br>3′-G.U.C.G.U.U.C.C.U.G.A*C*C*A*G*A.A.A.G.A.U-5′
B2M2-S10	5′-G.C.A.A.G.G.A.C.U.G.G.U.C.U.U.U.C.U.A.U.C-3′<br>3′-G.U.C.G.U.U*C*C*U*G*A.C.C.A.G.A.A.A.G.A.U-5′
B2M2-S11	5′-G.C.A.A.G.G.A.C.U.G.G.U.C.U.U.U.C.U.A.U.C-3′<br>3′-G*U*C*G*U*U.C.C.U.G.A.C.C.A.G.A.A.A.G.A.U-5′

Uppercase, unmodified RNA; bold lowercase, 2'-*O*-methyl RNA; asterisk, phosphorothioate linkage.

Supplementary Table 5. Cationic oligosaccharides extend the half-life of RNAs in serum. HP2 and HP2-S1 with or without the indicated oligosaccharides were incubated in 10% serum for 0 to 72 h. The remaining RNAs were quantified, and their half-lives were calculated. Each value is the mean ± SE (n = 4).

RNA	Oligosaccharide	Half-life (h)
HP2	None	5.79 ± 0.19
	ODAGlc4	8.74 ± 0.34
	ODAMan4	9.72 ± 0.24
	ODAGal4	12.2 ± 0.5
	ODGGal3	12.2 ± 0.7
HP2-S1	None	7.64 ± 0.06
	ODAGlc4	26.1 ± 1.9
	ODAMan4	29.6 ± 1.3
	ODAGal4	41.1 ± 1.2
	ODGGal3	40.0 ± 2.8

Supplementary Table 6. Sequences of single-stranded RNAs.

Name	*(S)/(AS)*	Sequence
(Unmodified)		
HP2-a	(*S*)	5′-CCAGUCAACAGGGGACAUAAA-3′
HP2-b	(*AS*)	3′-CUGGUCAGUUGUCCCCUGUAU-5′
B2M2-a	(*S*)	5′-GCAAGGACUGGUCUUUCUAUC-3′
B2M2-b	(*AS*)	3′-GUCGUUCCUGACCAGAAAGAU-5′
(Phosphorothioate modification)		
HP2-S-a	(*S*)	5′-C*C*A*G*U*C*A*A*C*A*G*G*G*G*A*C*A*U*A*A*A-3′
HP2-S-b	(*AS*)	3′-C*U*G*G*U*C*A*G*U*U*G*U*C*C*C*C*U*G*U*A*U-5′
B2M2-S-a	(*S*)	5′-G*C*A*A*G*G*A*C*U*G*G*U*C*U*U*U*C*U*A*U*C-3′
B2M2-S-b	(*AS*)	3′-G*U*C*G*U*U*C*C*U*G*A*C*C*A*G*A*A*A*G*A*U-5′
(2'-*O*-Methyl modification)		
HP2-M-a	(*S*)	5′-**ccagucaacaggggacauaaa**-3′
B2M2-M-a	(*S*)	5′-**gcaaggacuggucuuucuauc**-3′

Uppercase, unmodified RNA; bold lowercase, 2'-*O*-methyl RNA; asterisk, phosphorothioate linkage; (*S*), sense strand; (*AS*), antisense strand.

Supplementary Table 7. Sequences of double-stranded RNA 12mers.

Name	Sequence
12M-1	5′-A.C.U.G.A.C.U.G.A.C.U.G-3′<br>3′-U.G.A.C.U.G.A.C.U.G.A.C-5′
12M-S1	5′-A.C.U.G.A.C.U.G.A.C.U.G-3′<br>3′-U*G*A*C*U*G*A*C*U*G*A*C-5′
FAM-12M-1	5′-FAM-A.C.U.G.A.C.U.G.A.C.U.G-3′<br>3′- U.G.A.C.U.G.A.C.U.G.A.C-5′
FAM-12M-S1	5′-FAM-A.C.U.G.A.C.U.G.A.C.U.G-3′<br>3′- U*G*A*C*U*G*A*C*U*G*A*C-5′

Uppercase, unmodified RNA; asterisk, phosphorothioate linkage; FAM, 6-carboxyfluorescein.

(A)

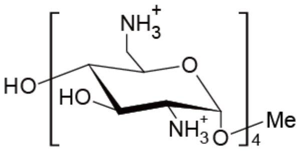

Oligodiaminoglucose 4mer (ODAGlc4)

(B)

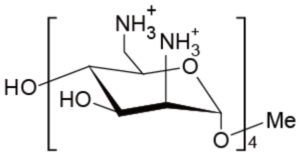

Oligodiaminomannose 4mer (ODAMan4)

(C)

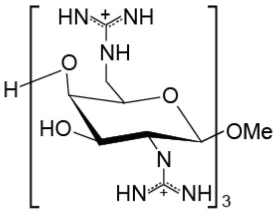

Oligodiguanidinogalactose 3mer (ODGGal3)

Supplementary Figure 1

(A)

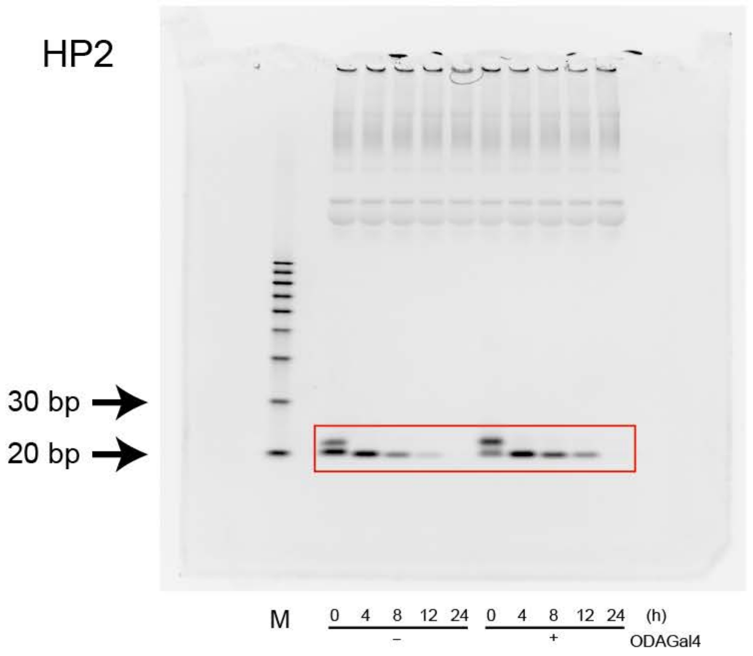

(B)

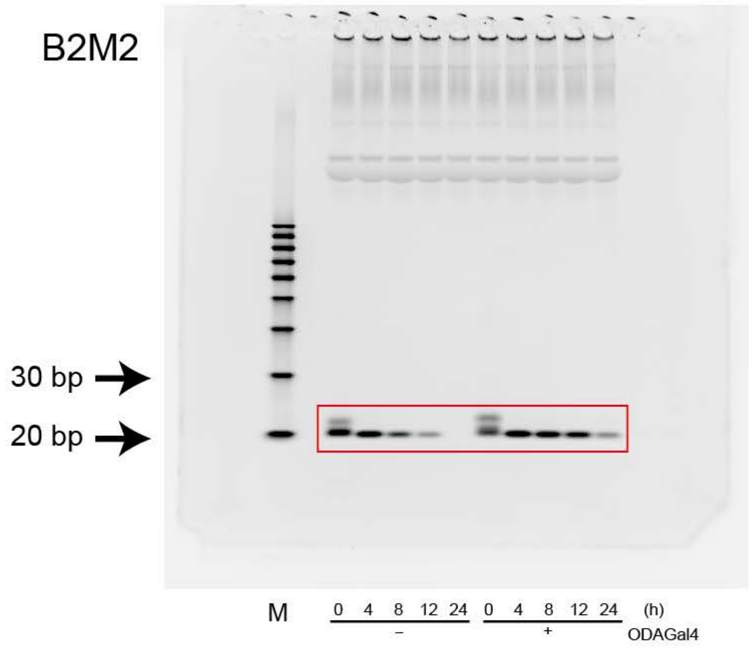

Supplementary Figure 2

(A)

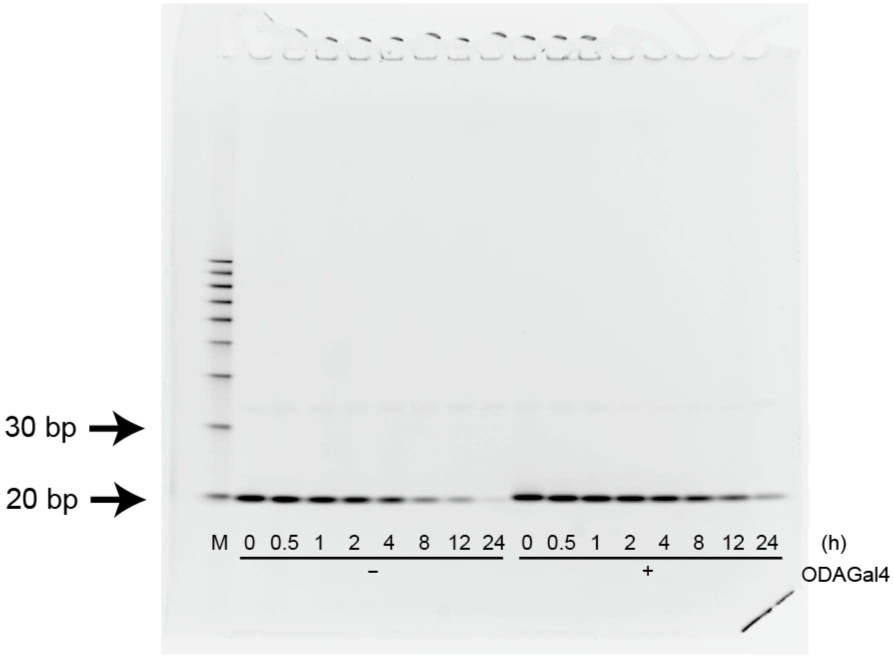

(B)

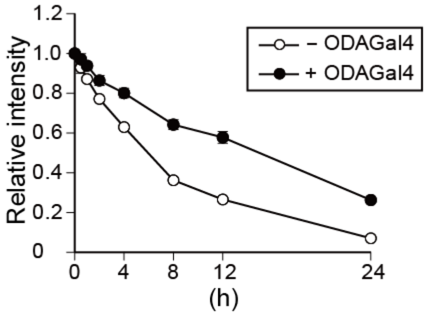

(C)

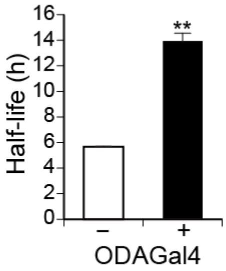

Supplementrary Figure 3

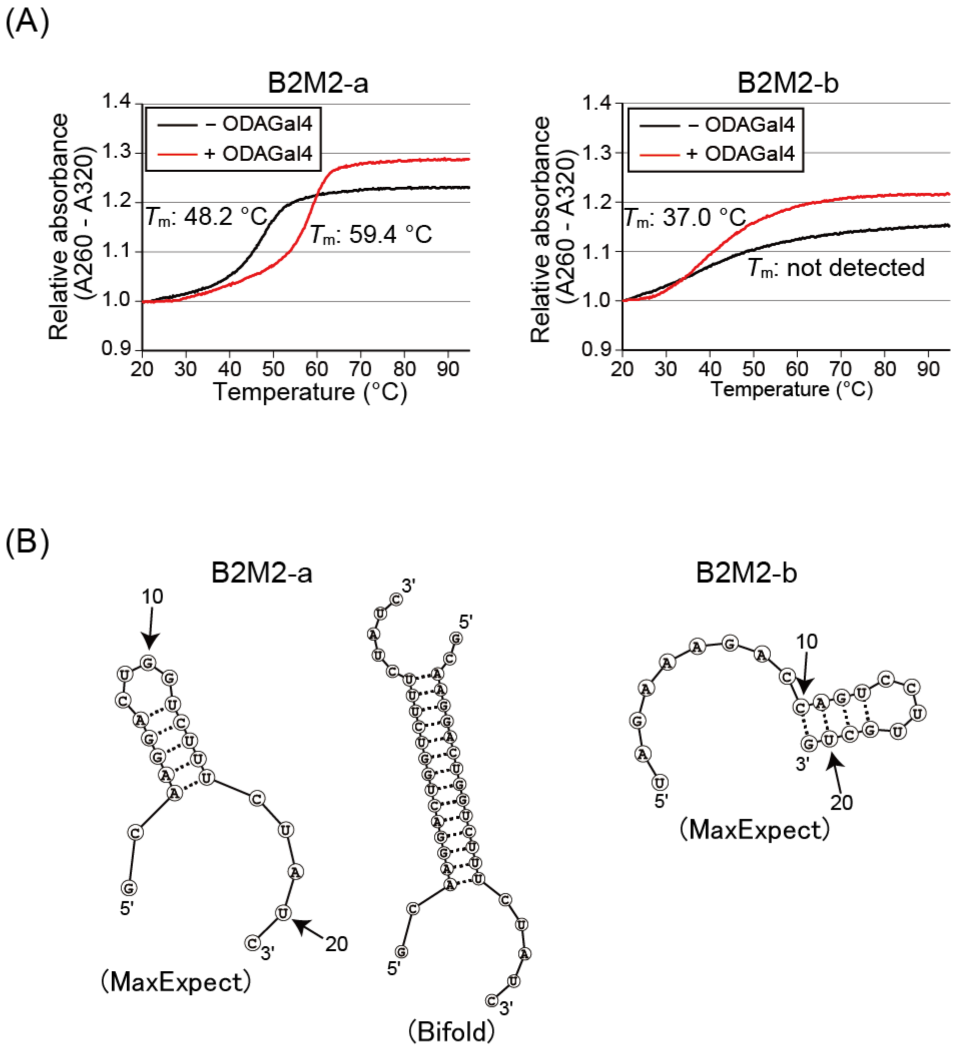

Supplementary Figure 4

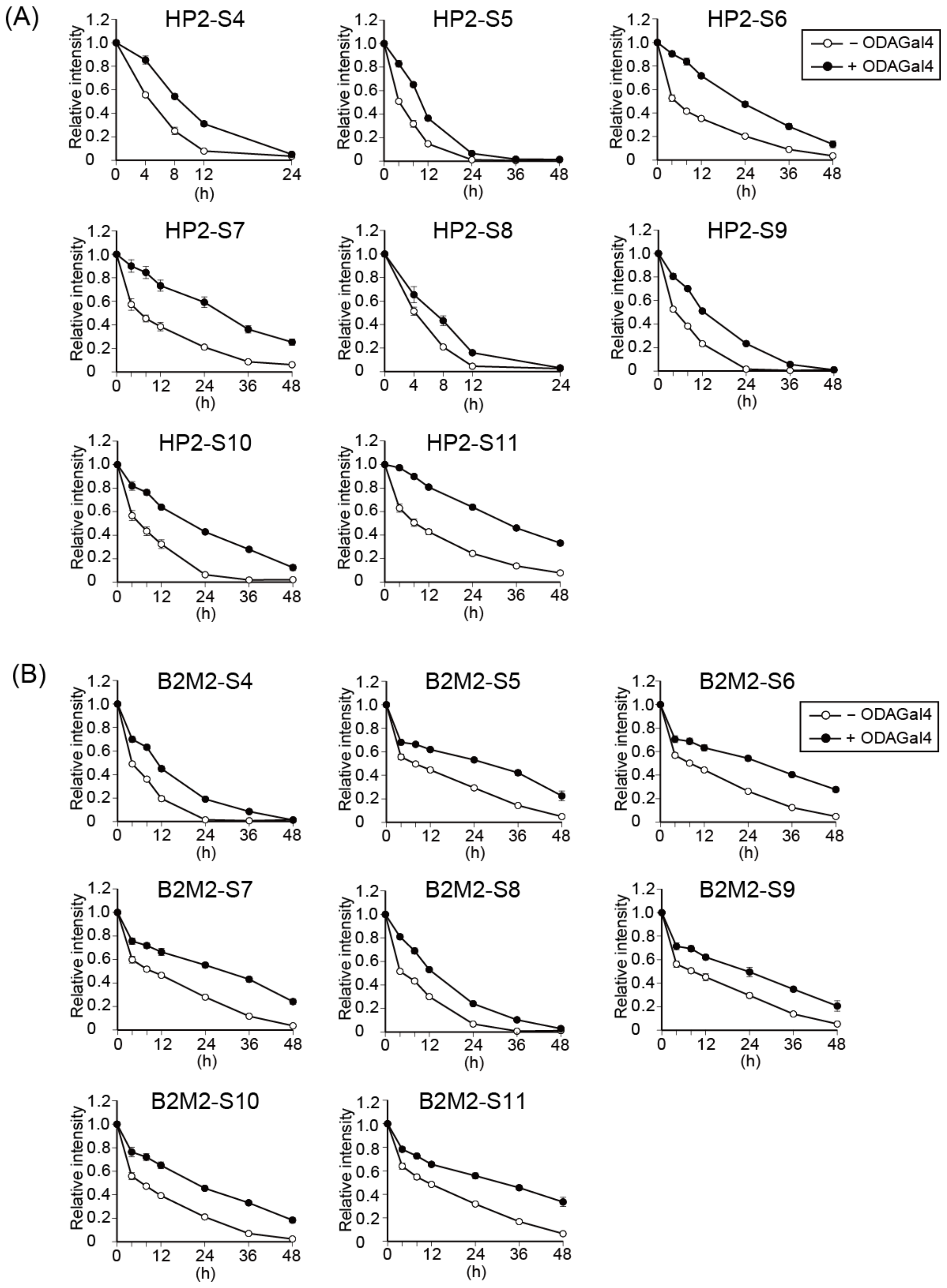

Supplementray Figure 5

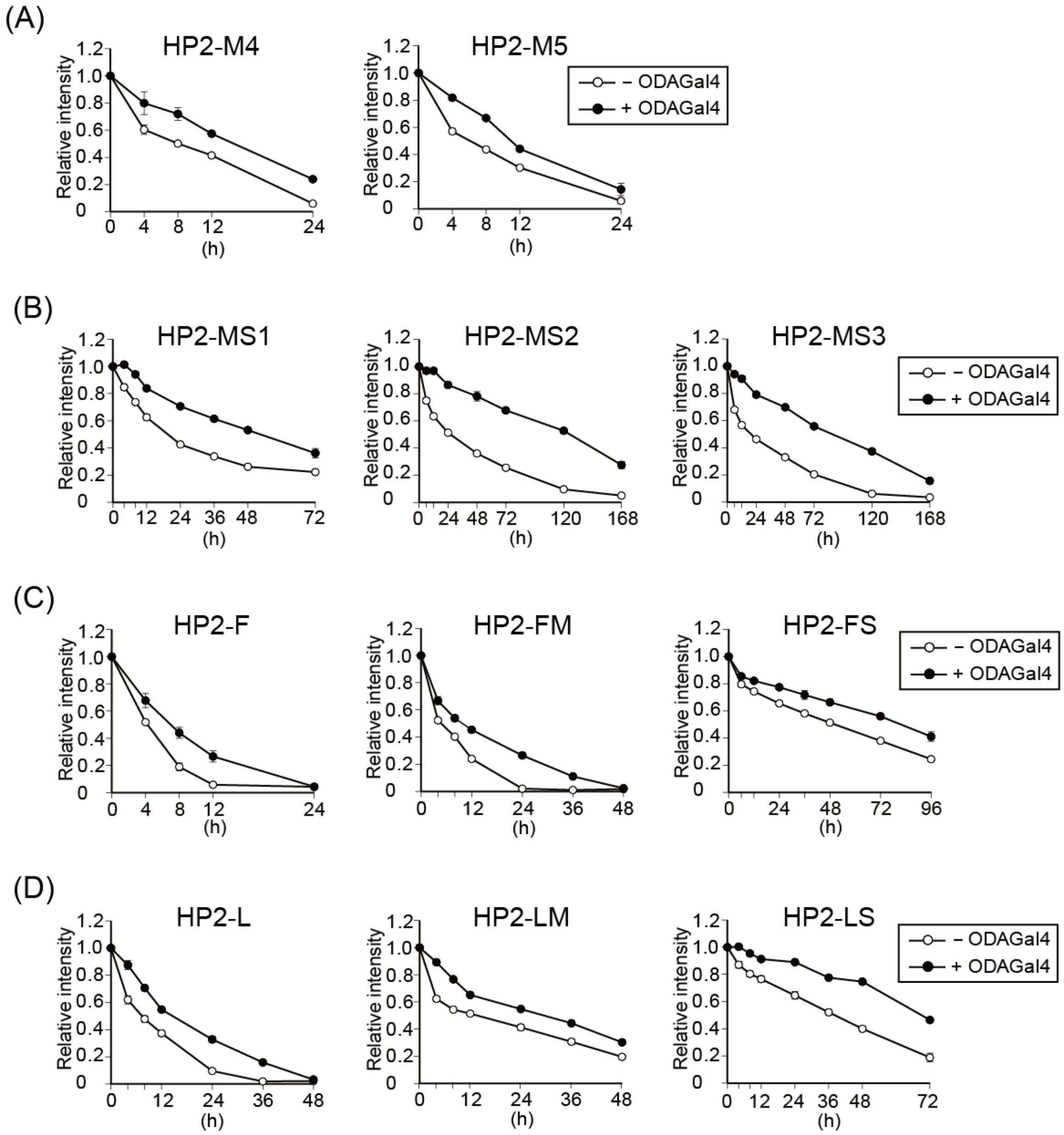

Supplementray Figure 6

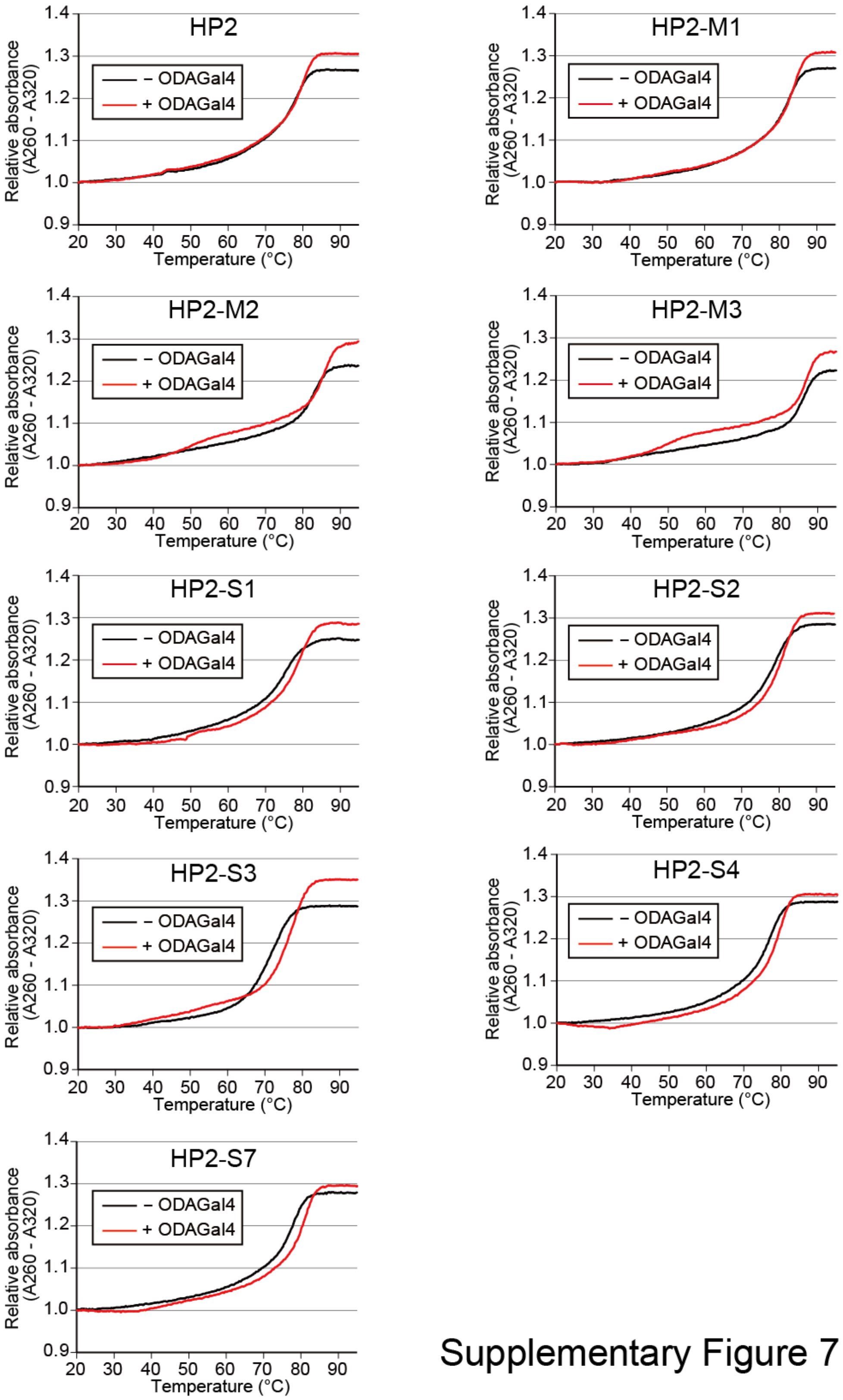

Supplementary Figure 7

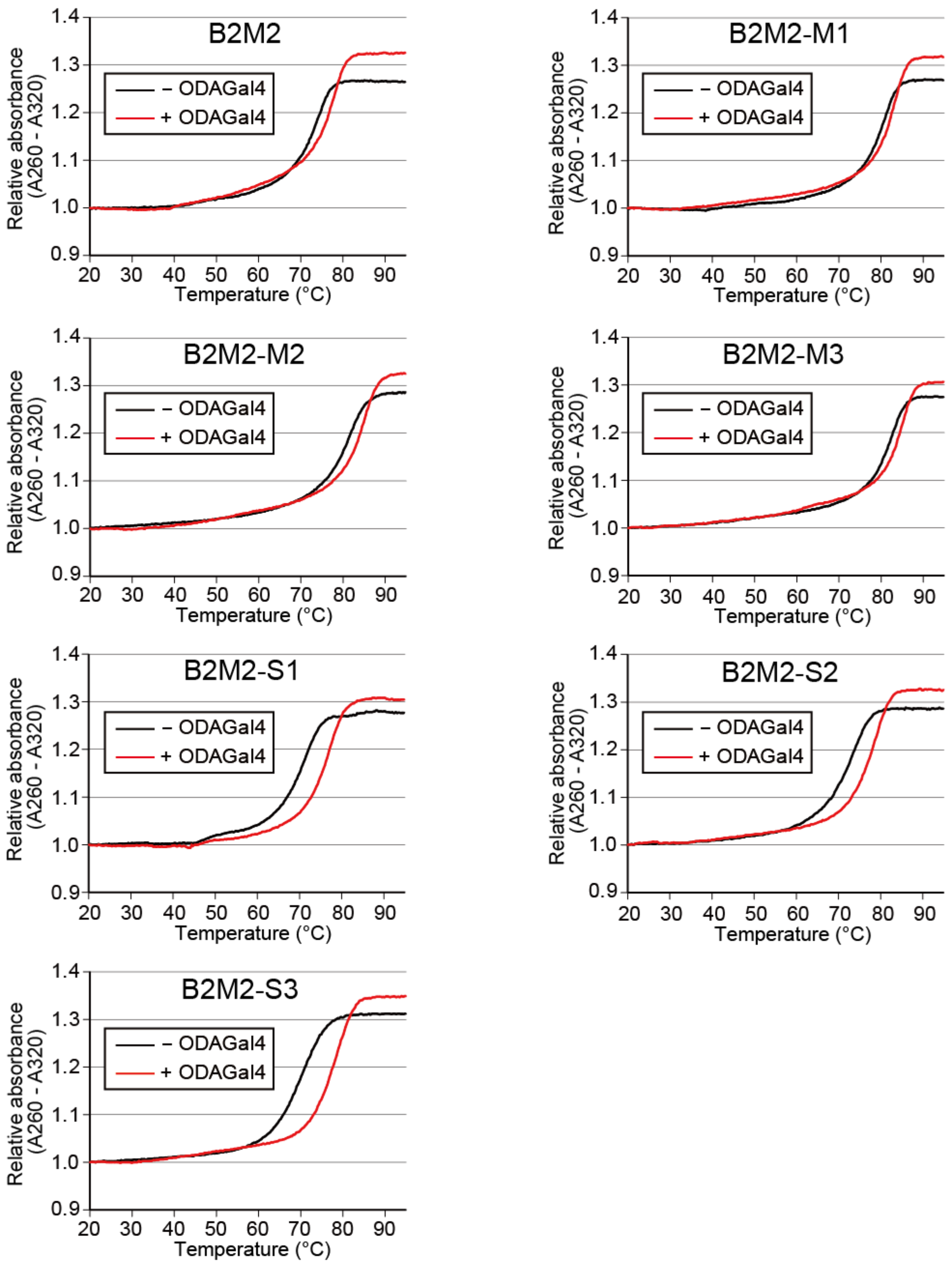

Supplementary Figure 8

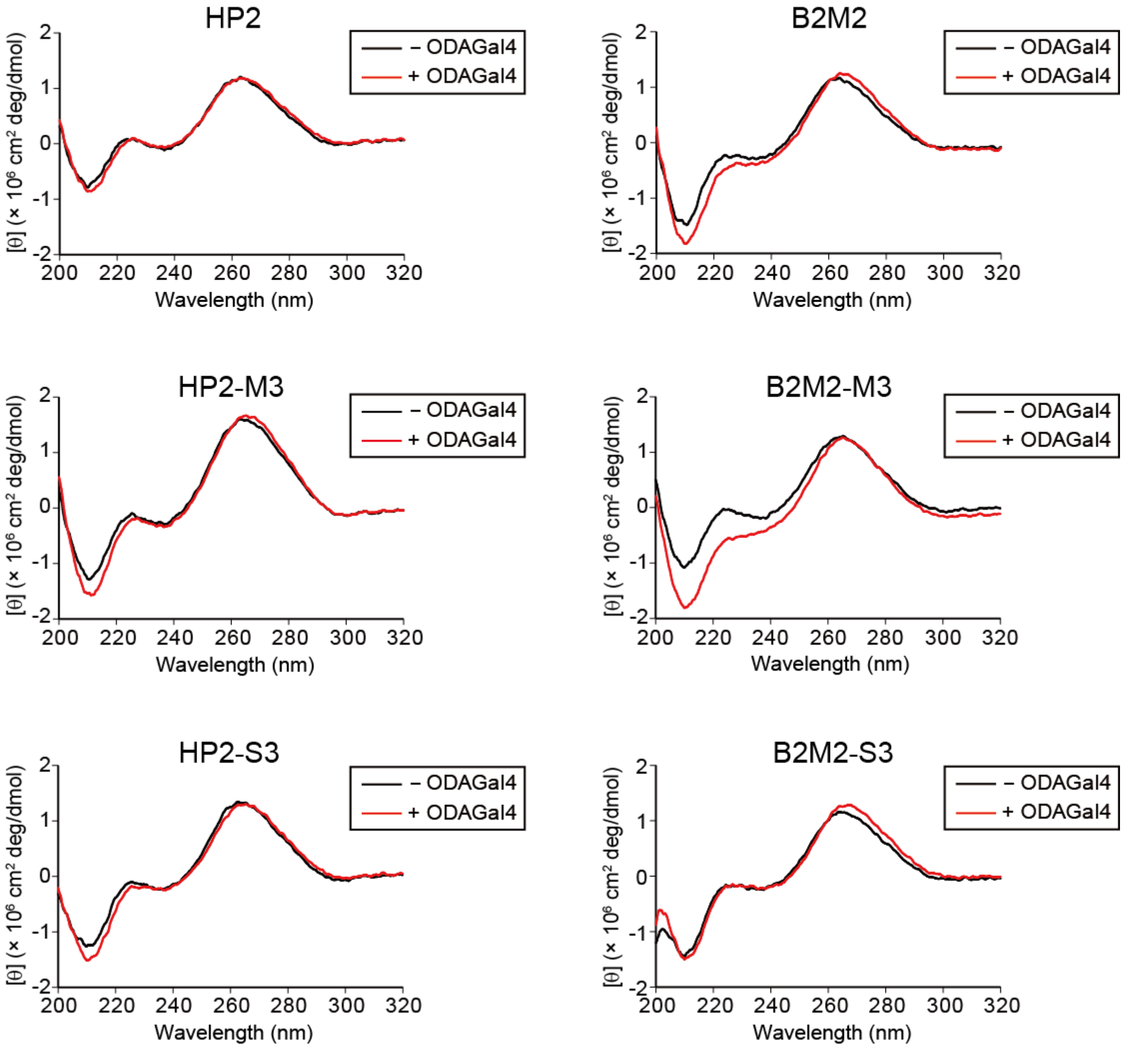

Supplementary Figure 9

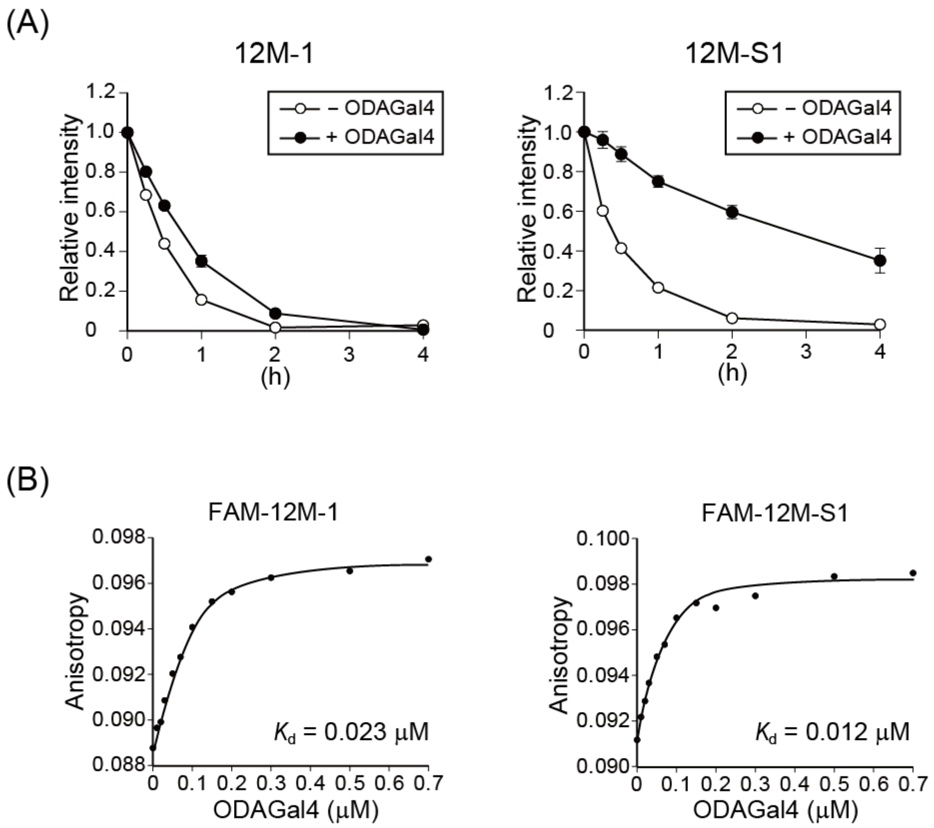

Supplementary Figure 10

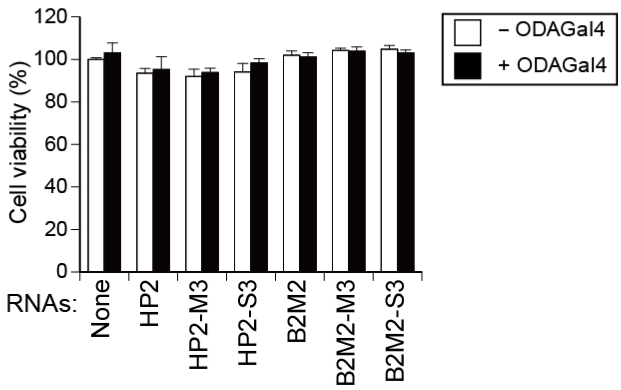

Supplementary Figure 11
